# A graph neural network framework for causal inference in brain networks

**DOI:** 10.1038/s41598-021-87411-8

**Published:** 2021-04-13

**Authors:** S. Wein, W. M. Malloni, A. M. Tomé, S. M. Frank, G. -I. Henze, S. Wüst, M. W. Greenlee, E. W. Lang

**Affiliations:** 1 CIML, Biophysics, University of Regensburg, 93040 Regensburg, Germany; 2 Experimental Psychology, University of Regensburg, 93040 Regensburg, Germany; 3 IEETA/DETI, Universidade de Aveiro, 3810-193 Aveiro, Portugal; 4 Department of Cognitive, Linguistic,and Psychological Sciences, Brown University, Providence, RI 02912 USA

**Keywords:** Dynamical systems, Network models, Computational science

## Abstract

A central question in neuroscience is how self-organizing dynamic interactions in the brain emerge on their relatively static structural backbone. Due to the complexity of spatial and temporal dependencies between different brain areas, fully comprehending the interplay between structure and function is still challenging and an area of intense research. In this paper we present a graph neural network (GNN) framework, to describe functional interactions based on the structural anatomical layout. A GNN allows us to process graph-structured spatio-temporal signals, providing a possibility to combine structural information derived from diffusion tensor imaging (DTI) with temporal neural activity profiles, like that observed in functional magnetic resonance imaging (fMRI). Moreover, dynamic interactions between different brain regions discovered by this data-driven approach can provide a multi-modal measure of causal connectivity strength. We assess the proposed model’s accuracy by evaluating its capabilities to replicate empirically observed neural activation profiles, and compare the performance to those of a vector auto regression (VAR), like that typically used in Granger causality. We show that GNNs are able to capture long-term dependencies in data and also computationally scale up to the analysis of large-scale networks. Finally we confirm that features learned by a GNN can generalize across MRI scanner types and acquisition protocols, by demonstrating that the performance on small datasets can be improved by pre-training the GNN on data from an earlier study. We conclude that the proposed multi-modal GNN framework can provide a novel perspective on the structure-function relationship in the brain. Accordingly this approach appears to be promising for the characterization of the information flow in brain networks.

## Introduction

Brain connectivity comes in different flavors, either resting on the structural anatomical layout, as derived from diffusion tensor imaging (DTI) or based on temporally resolved activity patterns, like observed in functional MRI (fMRI)^1^. White matter tracks reconstructed from DTI provide a foundation for structural connectivity (SC) and can be used to quantify the (static) anatomical connection strength between brain regions. On the other hand fMRI enables us to map out dynamic neural activity distributions across the brain, whereas the coherence of fluctuations is usually referred to as functional connectivity (FC). Such functional states can alternate very rapidly in contrast to changes in the structural connectome, alterations of which are mainly related to the natural development of the brain, aging or disease^2,3^. Therefore the brain structure can be considered as static during a fMRI measurement, in comparison to the fast functional fluctuations. Intuitively one might follow then the motto *“structure determines function”*, but it has been shown that the relationship between brain structure and function is quite complex and still a focus of intense research^4–8^. For instance, brain regions with robust SC usually show also high FC, but the inverse is not necessarily true^9^. While FC is a statistical measure with no information concerning the directionality of the relation, effective connectivity and directed functional connectivity measures try to infer causal dependencies in functional imaging data^10^. Thus connectivity measures derived from different modalities can provide distinct, but complementary aspects of brain connectivity^11–13^. Still, studying their relations is challenging mainly due to the complex spatio-temporal dependencies and inherent difficulty in long term forecasting.

In this paper we propose a data driven model, which combines information from fMRI and DTI to infer causal dependencies between brain regions. Temporal activity patterns of neuron pools, interconnected by the spatial anatomical layout, can be interpreted as time-varying graph structured signals. For such applications, graph neural networks (GNN) have shown to be useful, providing a possibility to process data with graph-like properties in the framework of artificial neural networks (ANN)^14^. Motivated by their success in computer vision^15,16^, convolution operations were recently extended to the graph domain^17,18^. Training such convolution filters in ANN enables us to capture inherent spatial dependencies in the non-Euclidean geometry of graphs, which are used in our context to integrate spatial relations of brain networks, based on their structural anatomical connections. Further, temporal dependencies in a dynamic system can be acquired by recurrent neural networks (RNNs) that have proven to be well suited for processing data with sequential structure. In our study, RNNs learn temporal characteristics of brain dynamics, like those observed in resting-state fMRI. A certain type of GNN architecture denoted as *diffusion convolution recurrent neural network* (DCRNN)^19^, provides the possibility to integrate spatio-temporal information of graph-structured signals. By combining fMRI with DTI data, the idea is to replicate brain dynamics more accurately, to get an improved understanding of functional interactions between brain regions, which are physically constrained by their structural backbone^20^.

Causal relationships between brain regions can be revealed by directed functional connectivity and effective connectivity. Two prominent and distinct approaches have been established in recent years^10^. The first one is based on a simple idea taken up by the British econometrician Clive Granger^21^. If one event *A* causes another event *B*, then *A* would precede *B*, and information on the occurrence of *A* should contribute to the prediction of the occurrence of event *B*. Such temporal dependencies between multivariate processes are typically described in the framework of a multivariate vector auto regressive (VAR) model, building a foundation for Granger causality (GC). By trying to make accurate predictions of temporal neural profiles, GC tests if adding information about neural activity in brain region *B* helps to improve the prediction of the activity in region *A* (and vice versa). This provides an exploratory measure for directed causal dependencies between segregated brain areas.

The second popular approach is methodologically different: Dynamic causal modeling (DCM) relies on a mechanistic input-state-output model of neuron pools, describing the effective connectivity strength between brain areas^22^. Experimental conditions and stimuli are encoded in input functions, and the model output can be related to empirically observed electromagnetic or hemodynamic responses. In a Bayesian framework, effective couplings of neural populations are estimated, providing a neurophysiological perspective on causal relationships between different regions in the brain. However due to its relatively high computational complexity, the analysis with DCM is usually limited to a few pre-defined regions in the brain only, what could neglect relevant components for the analysis^23^.

Here we present a data-driven machine learning approach that combines structural and functional information of neuron pools in a predictive framework for brain dynamics. By studying spatio-temporal dependencies between brain areas which were learned by the DCRNN model from DTI and fMRI data, we deduce the information flow between segregated areas in the brain. This provides us with a multi-modal data-driven perspective on causal relationships within brain networks. Currently, for investigating causal structures from an information theoretic perspective, a VAR is most often used as the underlying predictive model for Granger causality inference^10,24^. In our study we compare the VAR model to the multi-modal DCRNN model for this application. We test the capabilities of the two models to replicate empirically observed fMRI signals, in order to assess how well they can capture the underlying functional dynamics. First we show that the DCRNN is able to make more accurate long-term predictions in fMRI data. While a classical VAR model has to fit a parameter for each possible pair of the *N* brain areas in the network, which parameters then grow with an order $N^2$, the DCRNN learns localized filters on the structural network, also making its number of parameters independent of the network size^18^. This is especially useful for the analysis of large brain networks, when only sparse imaging data is available. Moreover, as white matter tracks build the physical substrate for the propagation of neural signals, a greater neurophysiological plausibility is provided by the DCRNN, because neural interactions are related to their anatomical connections in this model as well. This property makes such a GNN architecture also suitable for an explorative study of large-scale brain networks, unlike classical DCM, which is often limited to a few predefined brain regions only^23^.

Finally such a GNN framework, which integrates anatomical and functional neuroimaging data, can provide a novel perspective on the general relationship between brain structure and function. Until now different computational models have contributed valuable insights into the complex structure–function relationship, by simulating how empirically observed FC patterns can emerge from the structural backbone^4,6,9,25^. Furthermore, by relying on methods from graph theory, functional networks have been derived from a mapping of the underlying structural graphs^26–28^. Also approaches from machine learning have been employed to predict the strength of functional connections from the anatomical connectivity^29,30^, and more recently some models have been proposed that additionally considered dynamic FC patterns for studying this relation^20,31^.

So far most of the methods for investigating the structure–function relationship try to predict only the temporal coherence patterns of functional fluctuations (FC) from their structural connectivity profile (SC), but such a correlation based FC might be limited in its ability to characterize the rich nature of functional brain dynamics. Instead of replicating only FC patterns, our approach directly models the measured neural activity in different brain areas, thereby capturing more of the original information in the empirically observed functional dynamics. By modeling neural interactions on the structural backbone, this method allows us to reconstruct the amount of information on activity distributions that occurs in structurally connected brain regions. Accordingly, such a spatio-temporal GNN model can provide a novel possibility to investigate the complex structure-function relation under a different lens.

Usually for a good performance more complex machine learning models require a larger amount of data, but it is not economical feasible in MRI to perform studies with very large sample sizes. To account for these issues we demonstrate that also in our context transfer learning^32^ can enhance the model accuracy of small datasets. We pre-train the DCRNN on a large-scale dataset of 100 resting-state fMRI (rs-fMRI) sessions provided by the Human Connectome Project^33^ (HCP). We then show that the pre-trained model considerably improves the predictive performance on a smaller independent dataset of 10 sessions compared to standard training. This points to the ability of the DCRNN to generalize across scanner types and acquisition protocols to a certain extent, enabling the possibility for transfer learning.

## Results

### Model description

**Figure 1:**
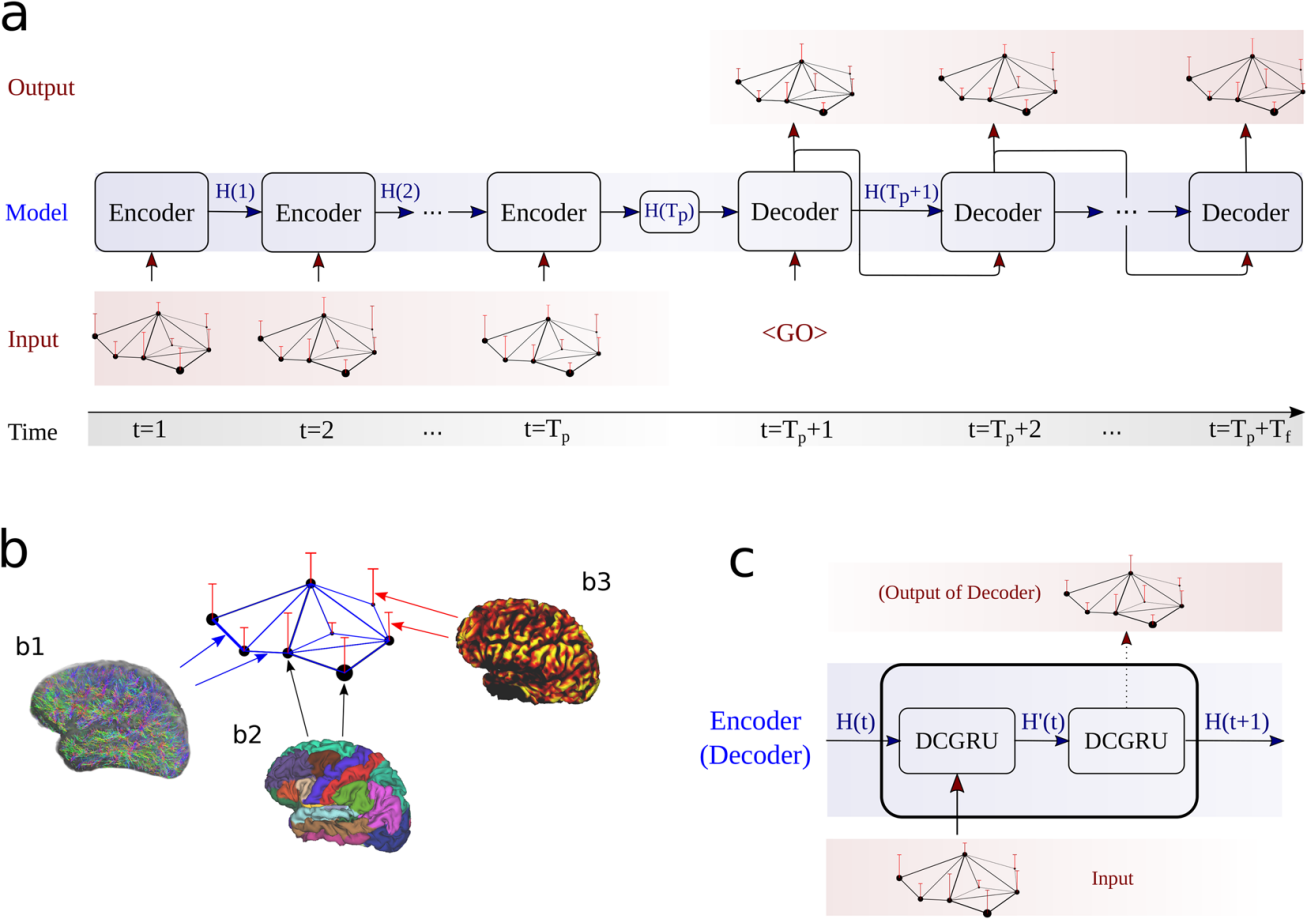
An overview of the DCRNN model. The model consists of an encoder and decoder (**a**), modified to process graph structured signals (**b**). In our context, vertices (nodes) ${\mathcal {V}}, |{\mathcal {V}}| = N$ of the graph $\mathcal {G}$ are defined as *N* brain regions, derived from an atlas (**b2**). Structural connections between brain regions are derived from DTI, quantifying the strength of edge connections in the graph (**b1**). The signal on the graph ${\mathbf {x}}(t)$ at a certain time point *t* is the average BOLD signal in brain regions/nodes, obtained by the fMRI measurement at time *t* (**b3**). The encoder (**a**) receives an input sequence $[{\mathbf {x}}(1),\ldots ,{\mathbf {x}}(T_p)]$, and iteratively updates its hidden state *H*(*t*). The final encoder state $H(T_p)$ is passed to the decoder part, which learns to recursively predict the output sequence of graph signals $[{\mathbf {x}}(T_p+1),\ldots ,{\mathbf {x}}(T_p + T_f)]$ in the future. The encoder, as well as the decoder (**c**) consist of diffusion convolution gated recurrent unit cells (DCGRU). The first encoder and decoder cell receive the input graph signal, and they pass their hidden state to the subsequent cell. In the decoding part, the final cell of the decoder generates then the predicted signal (**c**). During testing and validation, the decoder uses its own outputs as inputs, to generate the subsequent output. The first input of the decoder ($<GO>$ symbol) is simply a vector of zeros. Figure (**b1**) was created with the *MRtrix3* software package^35^ (version 3.0) : https://www.mrtrix.org/, and figure (**b2**) and (**b3**) with the Connectome Workbench (version 1.4.2): https://www.humanconnectome.org/software/connectome-workbench.

In this study we use the DCRNN model^19^ architecture to explore the spatio-temporal relationships of brain dynamics in resting state fMRI. An overview of the model structure is provided in Fig. 1. To learn the temporal dependencies of the BOLD signal, recurrent neural networks (RNNs) with sequence-to-sequence learning are employed^34^. In such an architecture the encoder network maps information from an input sequence into a hidden representation, which is used by the decoding part to sequentially generate outputs, based on this encoded information. In the context of brain dynamics, the input sequence corresponds to measurements of the BOLD signal ${\mathbf {x}}(t) \in {\mathbb {R}}^{N}$ in *N* brain regions at $T_p$ time points, while the objective is to predict the signal at $T_f$ subsequent time points.

In addition to temporal, also spatial dependencies between brain regions are incorporated via diffusion convolution operations^19^. Consider the network of regions of interest (ROIs) as a *graph* $\mathcal {G} = ({\mathcal {V}}, \mathcal {E},{{\mathbf {A}}}_w)$, where ${\mathcal {V}}, |{\mathcal {V}}| = N$ denotes a set of vertices (nodes), $\mathcal {E}$ represents a set of edges and ${{\mathbf {A}}}_w \in {\mathbb {R}}^{N \times N}$ is a *weighted adjacency matrix*. The latter represents the spatial connectivity of the nodes, i.e. the ROIs on the neuronal network, which are adjacent to each other, i.e. connected by an edge. Also the weights result from DTI, reflecting the axonal connection strength between the connected regions. Goal of the DCRNN model is to learn a function *h*(...) which maps $T_p$ past activity states ${\mathbf {x}}(t)$, to $T_f$ future states:

1 $$\begin{aligned} \left[ {\mathbf {x}}(t-T_p+1), \ldots , {\mathbf {x}}(t); \mathcal {G}\right] \xrightarrow {h(...)} \left[ {\mathbf {x}}(t+1), \ldots , {\mathbf {x}}\left( t+T_f\right) \right] . \end{aligned}$$

The encoder, as well as the decoder of the DCRNN consist of gated recurrent units^36^, modified with graph convolutions^18^, and for training the model scheduled sampling was applied^37^. A detailed description of the model architecture is provided in the methods section.

### Data description

For the first part of our evaluation, resting-state fMRI data from the *S1200 release* provided by the *Human Connectome Project*^33^ (HCP) was employed^38^. Further the multi-model parcellation proposed by Glasser et. al^39^ was applied to divide each hemisphere into 180 segregated regions. The BOLD signal in each region was averaged, so for each resting state session, $N=360$ time courses were obtained. During each session $T=1200$ images were acquired, so the data can be arranged in a matrix ${\mathbf {X}} \in {\mathbb {R}}^{N \times T}$. For the following analysis, we filter the data with a 0.04–0.07 Hz narrow band bandpass filter, as it has shown to be reliable and functionally relevant for gray matter activity^20,40–43^. We additionally present results in supplement IV, when employing a more liberal bandpass filter with cutoff frequencies between 0.02 and 0.09 Hz.

The input and output (label) samples for the DCRNN model were generated from the data in ${\mathbf {X}}$, by defining windows of length $T_p$ to obtain input sequences of neural activity states $[{\mathbf {x}}(t-T_p+1), \ldots , {\mathbf {x}}(t)]$, and respective target sequences $[{\mathbf {x}}(t+1), \ldots , {\mathbf {x}}(t+T_f)]$ of length $T_f$. The index *t* was propagated through each resting-state fMRI session, so in total $T - T_p - T_f + 1$ input-output pairs were generated per session. The first $80\%$ of those time window samples of each fMRI session were used for training the DCRNN model, the subsequent $10\%$ for validation, and the last $10\%$ for testing. In total 4 resting-state fMRI sessions from 25 different subjects were employed for the evaluations in the following sections. The input and output length was chosen to be $T_p = T_f = 30$, what would correspond to a time span of roughly $22\ s$, based on the sampling with a repetition time $TR = 0.72\ s$^44^. But note that in general the sequence-to-sequence architecture employed would be able to deal with arbitrary input and output signal lengths^34^.

In addition to temporal brain dynamics, also structural information was incorporated into the model, described by the anatomical connection strength between brain regions deduced from DTI. Therefore the DTI dataset provided in the *S1200 release*^38^ was further processed by employing multi-shell, multi-tissue constrained spherical deconvolution^45^, implemented in the *MRtrix3* software package^35^. White matter tractography was performed to estimate whole brain structural connectivity between the $N=360$ regions of the multi-modal parcellation atlas^39^. The number of generated streamlines connecting two brain regions were used to define the edge strengths in the graph adjacency matrix ${{\mathbf {A}}}_w \in {\mathbb {R}}^{N \times N}$. A more detailed description of the datasets and preprocessing involved can be found in the methods subsection ‘HCP data’.

### Model performance

In a first step we assess the capabilities of the DCRNN model to learn temporal activity patterns in neuron pools, and their relationships across the spatial layout. As a first baseline we compare the DCRNN to the performance of a linear vector autoregressive (VAR) model^21^, further described in the subsection ‘Autoregressive models’. A common way to estimate causal relations between different regions of interest (ROIs) in a brain network, is to fit a multivariate VAR model to neural temporal activity patterns, like those observed in different neuroimaging modalities^10,24,46^. Evaluating the fitted VAR allows us to infer, if one spatial brain region, contains additional information about future activity profiles of other regions, indicating a causal dependency between them. The accuracy in replicating empirically observed neural activity profiles can indicate how well a model has learned the underlying process of neural dynamics, including the interactions and dependencies among brain regions. In this comparison we incorporated two different optimization methods for the estimation of the VAR coefficients. The first one employs an ordinary least squares (OLS) fit on the neural activity timecourses ${\mathbf {x}}(t)$ from individual rs-fMRI sessions^47^. The second approach, in analogy to the DCRNN, follows a gradient-descent based optimization^48^ on the windowed neural activity samples as outlined in the subsection ‘Data description’. For this evaluation we rely on the latter one, as it could improve the performance of the VAR, as described in the subsection ‘Autoregressive models’.

**Figure 2:**
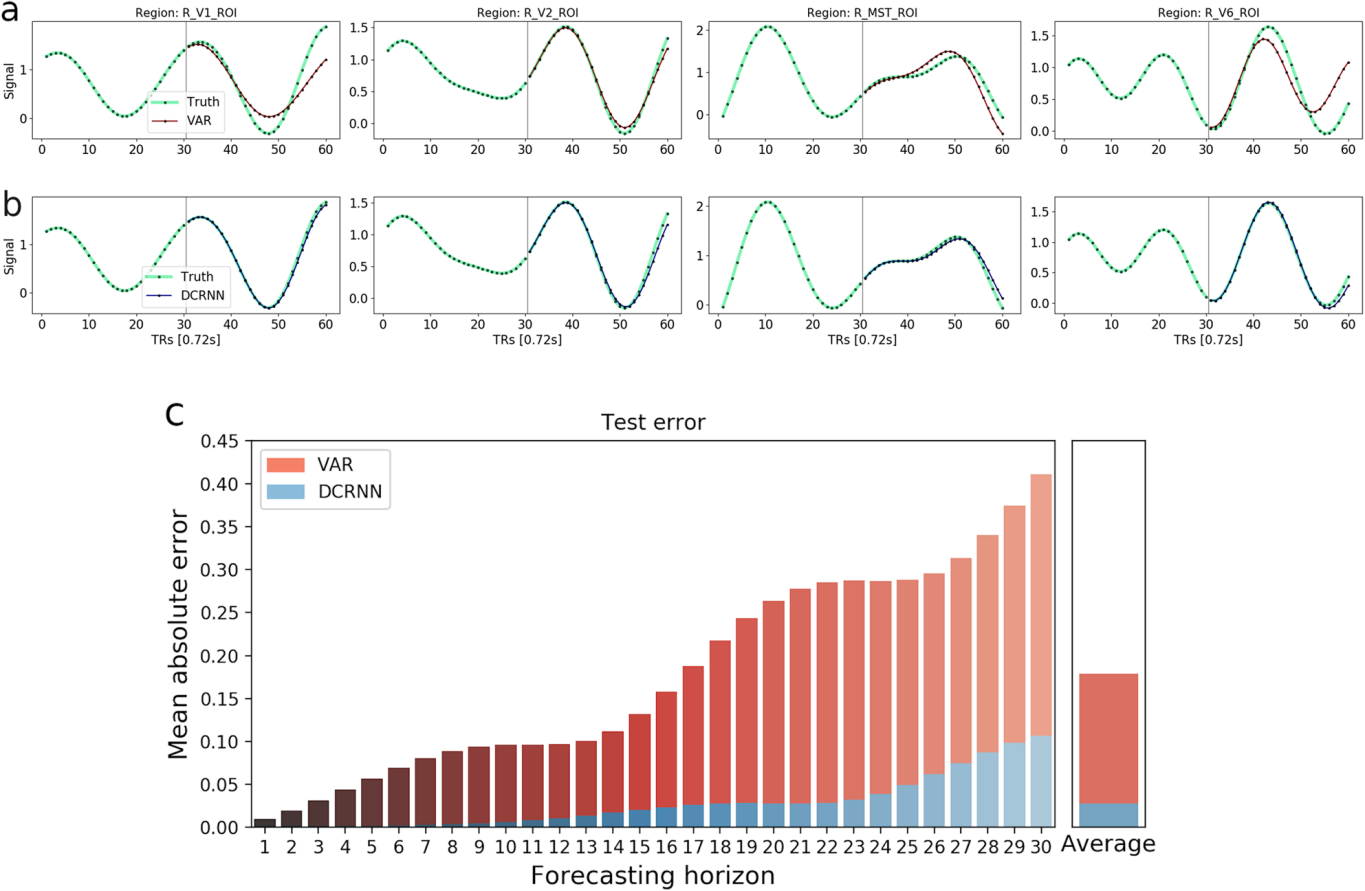
The figure illustrates the prediction accuracy of a VAR model (**a**) in comparison to the DCRNN (**b**). The true BOLD signal in these 4 ROIs is marked green, while predictions of the VAR are highlighted in red, and for the DCRNN in blue. The first $30 \,TRs$ of BOLD signal were used as the model inputs, and the goal was to predict the subsequent $30 \,TRs$. This illustrative example was chosen to represent the whole test set, the prediction error of the VAR model on this sample is 0.169, and as such slightly below average, while the error of the DCRNN is with 0.037 higher than its average. Below in (**c**) the overall test MAE is illustrated in dependence of the forecasting horizon, computed as the average over all subjects, sessions, brain regions and test samples. On the right side in (**c**) the average of all horizons is shown.

The evaluations were performed on test data from 100 rs-fMRI sessions (4 sessions from 25 subjects), using the last $10\%$ of the time window samples of each session, corresponding to 114 test samples per session. Within a time window length of $T_p = T_f = 30$ both models can make relatively reasonable predictions, but also the difference in the prediction accuracy becomes apparent. A representative example of the accuracy of the two approaches is shown in Fig. 2, as well as their average performance on the complete testing data set. Figure 2a illustrates that a linear VAR model can generate in a few cases also correct long term predictions, but most often after $10 \,TRs$ ($\approx 7$ s) the error starts to accumulate and the predictions become less accurate. The predictions of the DCRNN (Fig. 2b) remain stable over much longer forecasting horizons, and the average mean absolute error $MAE=0.0279$ is considerably lower than the $MAE=0.1786$ of the VAR.

To further test the significance of the performance difference across subjects, the overall MAE between the predicted and true BOLD signals were computed for each of the 25 subjects individually, as an average across sessions, brain regions and test samples. A paired t-test was applied and with considering a significance threshold of 0.05, the difference in forecasting accuracy between the models showed to be highly significant with $p \le 0.0001$ across subjects. Due to the oscillations of the BOLD signal, the predicted signals tend to intersect the true ones at some point in time, what can be seen for example in Fig. 2a. After the truth and prediction have diverged from each other at previous timepoints, at this point of intersection the MAE tends to be smaller again. Therewith these intersections are reflected in some flatspots occurring in the error bars along the forecasting horizon, like observed in Fig. 2c. The forecasting error in dependence of different model output horizons is provided in Table 1. The difference in the performance between the DCRNN and VAR model already becomes apparent within the first few predicted timesteps, and the margin tends to further increase for larger prediction horizons.

**Table 1:** The overall test MAE of the VAR and DCRNN model, in dependence of different forecasting horizons.

Model	Forcasting horizon (TRs)					
	5	10	15	20	25	30
VAR	0.0321	0.0589	0.0751	0.1099	0.1449	0.1786
DCRNN	0.0018	0.0028	0.0065	0.0115	0.0163	0.0279

Additional analysis of the performance of the DCRNN model can be found in the Supplementary Information. At first in supplement I we reproduced the evaluations by testing the prediction accuracy on a cohort of new and unseen subjects. Further in Supplementary I we have discussed the impact of the training dataset size, as well as the role of the input horizon length $T_p$ on the model’s prediction performance. Additionally we studied the consistency of the model performance across subjects and we investigated, how the prediction accuracy depends on the different brain regions, to examine if there are areas with more or less complex temporal dynamics. To test the efficiency of neural network architecture implemented by the DCRNN, we then compared it to different baseline models in Supplementary II. Further in Supplementary III we compared the VAR and DCRNN on a smaller dataset collected at a different imaging site. To study the impact of a more liberal frequency filtering within the 0.02–0.09 Hz range, the equivalent evaluation is provided in Supplementary IV. In Supplementary V we evaluate the different approaches employing the volumetric AAL parcellation^49^ and performing an alternative method for reconstructing the anatomical connectivity^50^. Finally the effects of the timeseries input length and optimization methods on the VAR model performance are discussed in Supplementary VI.

### Impact of spatial modeling

**Figure 3:**
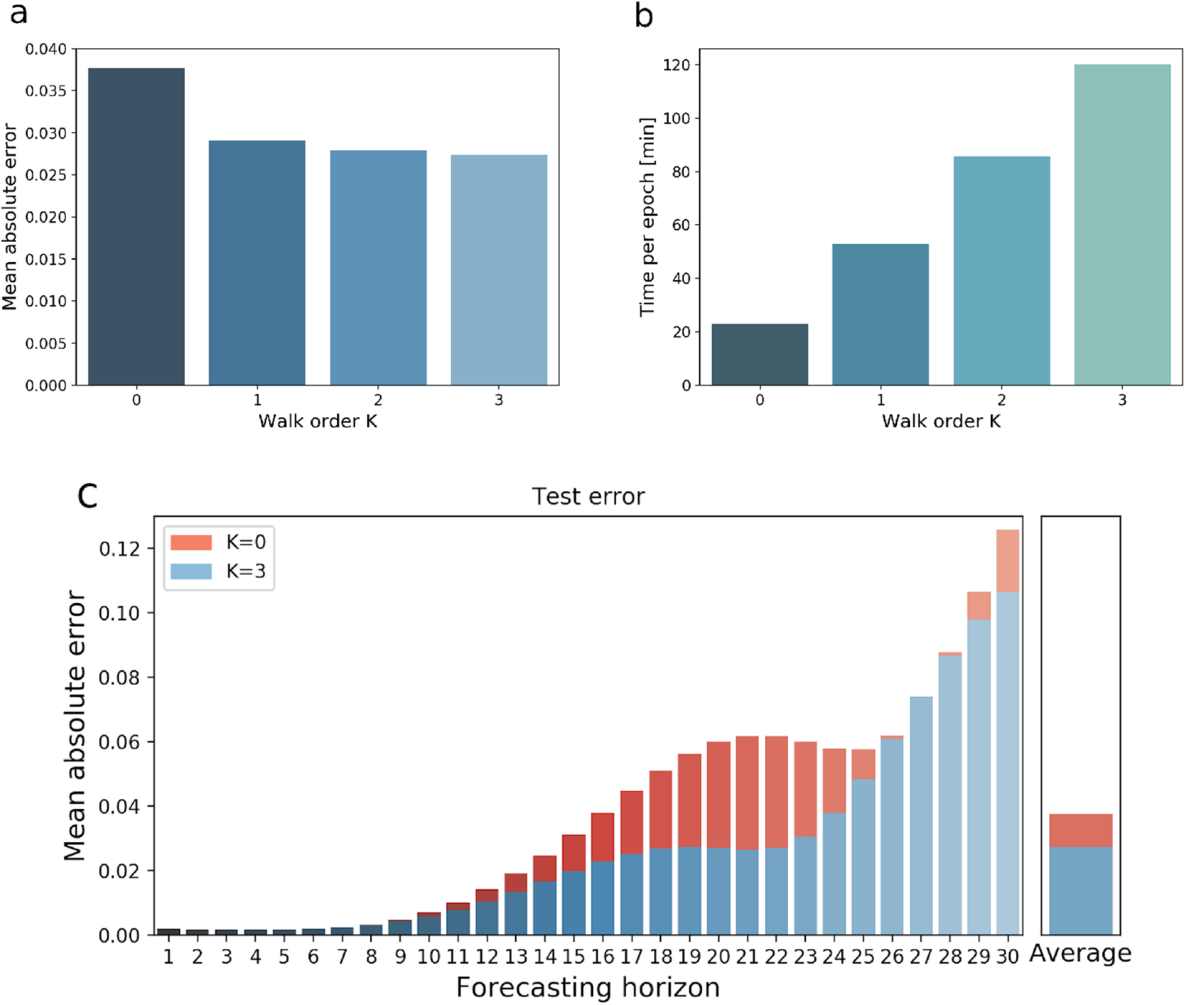
This figure depicts the effect of structural modeling on the prediction accuracy. The MAE values were computed as the average over all subjects, sessions, brain regions and test samples. In (**a**) the test MAE in dependence on included walk order *K* is shown, while (**b**) demonstrates the impact of *K* on the computational load per epoch. A more detailed comparison of the MAE on the forecasting horizon when employing filters with order $K=0$ and $K=3$ is illustrated in (**c**).

For this application of the DCRNN model, the anatomical connectivity was used to characterize spatial relations between nodes in the brain network, shaping the transition of activity between brain regions. To illustrate that the DCRNN indeed has learned relevant spatial interactions between different ROIs, we evaluate this recurrent neural network model, without employing graph (diffusion) convolution layers. This restriction considers only self-couplings (filters of order $K=0$) of nodes on the structural graph. Figure 3a shows the test MAE in dependence of the incorporated walk order *K*. The increase in computational time per epoch in dependence of included transition orders *K* is depicted in Fig. 3b. A more detailed comparison of the prediction MAE between the sequence-to-sequence model without graph convolutions ($K=0$), and including spatial transitions up to order $K=3$ is illustrated in Fig. 3c.

These results show, that the vast amount of the information about future activity in one region comes from the region itself. But by including first order transitions on the structural network ($K=1$) the error can already be decreased by $25\%$. Filters of higher orders $K=2,3$ only slightly improve the predictions further, as shown in Fig. 3a, but the computational load increases linearly with order *K*, like illustrated in Fig. 3b. The role of such transitions within the anatomical network can tell us something about the general structure–function relationship in the human brain, by pointing out how much information about functional dynamics comes from structurally connected regions. The comparison between $K=0$ and $K=3$ shows, that roughly up to $27\%$ of the predictive performance can be attributed to information from regions that are anatomically connected with each other. A paired t-test was applied to test the difference in the models accuracy between $K=0$ and $K=3$ across subjects, which turned out to be significant with a value of $p\le 0.0001$.

### Causal connectivity

In this section the objective is to study the principle of information passing between different ROIs the DCRNN has learned from the neuroimaging data. As shown in the previous subsection ‘Impact of spatial modeling’, propagating information on the anatomical network can improve the predictions of the temporal evolution of the BOLD signal, displaying a dependence among structurally connected brain regions. Such a dependency might go beyond simple coherency based functional connectivity, as the latter usually assess only the temporal similarity of two signals. Observing that the past activity in some regions contains additional information about the future activity in other regions, beyond information retrieved from their own past, could indicate some flow of information among them and could provide a first indication for a causal dependency structure. Now to derive such a measure of causal connectivity strength, by following the idea of Granger^21^, the goal is to reconstruct how information about the activity in ROI *A* contributes to the prediction of the activity in ROI *B*. To reveal relationships inside the data by directly looking at the learned parameters is often challenging when ANN models become more complex. One simple strategy used to account for this problem is to induce perturbations in the models input space and then observe how these perturbations are propagated to the models outputs^51,52^.

In our context, the DCRNN first learns a function *h*(...), mapping the original input sequences of neural activity states $[{\mathbf {x}}(t-T_p+1), \ldots , {\mathbf {x}}(t)]$ to a predicted output sequences of future states $[{{\hat{{\mathbf {x}}}}}(t+1), \ldots , {{\hat{{\mathbf {x}}}}}(t+T_f)]$. Then the information about the activity in a ROI $n'$ is removed, by simply replacing the values $x_{n'}(t)$ in the input sequence with the mean value of the data distribution $\bar{x}_{n'}(t) = 0$. Next the input sequence with the artificial perturbation in $n'$ is projected by the model *h*(...) to an output sequence $[{{\hat{{\mathbf {x}}}}}^\prime(t+1), \ldots , {{\hat{{\mathbf {x}}}}}^\prime(t+T_f)]$. Finally the differences of the models predictions ${{\hat{\mathbf{x}}}}'(t)$ with the perturbation in the input space in ROI $n'$, and the predictions ${{\hat{{\mathbf {x}}}}}(t)$ with the original input can be compared. A measure of influence ${\mathbf {I}}(n') \in {\mathbb {R}}^N$ of the information in ROI $n'$ on the predictions in other ROIs can then be defined as:

2 $$\begin{aligned} I_n(n') = \frac{1}{S} \sum _{s=0}^{S} \frac{1}{T_f} \sum _{t=0}^{T_f} | {{\hat{{\mathbf {x}}}}}_n^{(s)}(t) - {{{\hat{{\mathbf {x}}}}}_n}'^{(s)}(t) | \end{aligned}$$

with $I_n(n')$ describing the impact of region $n'$ on region *n*. Here ${{\hat{\mathbf{x}}}}_n^{(s)}(t)$ and ${{{\hat{\mathbf{x}}}}_n}'^{(s)}(t)$ denote the predictions in region *n* with and without the perturbation of $n'$ in the input space respectively, of a test sample *s* at time step *t*.

**Figure 4:**
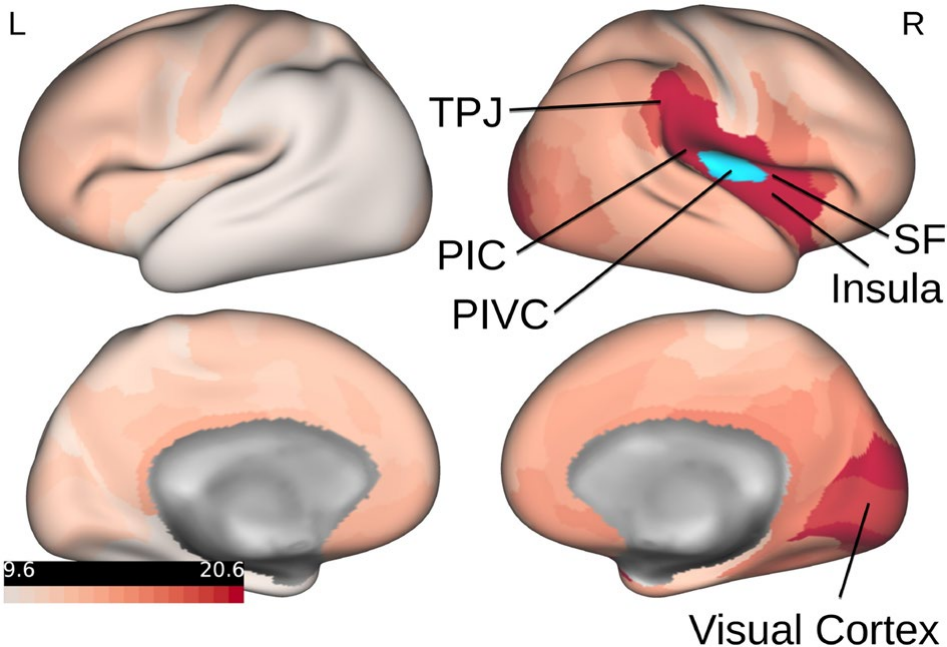
The figure illustrates the influence of activity in PIVC on all other brain regions. The left side depicts the left hemisphere, while on the right side the right hemisphere is shown. The target region PIVC in the right hemisphere is marked in blue. The values of the influence measure ${\mathbf {I}}(n')$ were normalized between 0 and 100 and are encoded in red in this illustration. *PIC* posterior insular cortex, *PIVC* parieto-insular vestibular cortex, *SF* Sylvian fissure and surrounding perisylvian cortex, *TPJ* temporo-parietal junction. Note that causal relationships from right PIVC were primarily found in the ipsilateral hemisphere. The figure was created with the Connectome Workbench software (version 1.4.2): https://www.humanconnectome.org/software/connectome-workbench.

To visualize this measure of influence of $n'$ on each individual region *n*, values of ${\mathbf {I}}(n')$ can be projected onto the cortical surface. In the following we studied the impact of the parieto-insular vestibular cortex (PIVC) on all other brain regions. Here PIVC in the right hemisphere is characterized as a conjunction of ROIs R_OP2-3 and R_Ig, as defined by Glasser et al.^39^. Previous results show that this location coincides with the average location of PIVC across human subjects^53,54^. The perturbation was induced in R_OP2-3 and R_Ig simultaneously, and Fig. 4 illustrates the strength of influence on all other regions (encoded in red) of the target region PIVC (marked in blue).

The results of this analysis show that PIVC exhibited an interrelationship with the Sylvian fissure, the perisylvian cortex and the insula. Similar connectivity patterns have been observed using diffusion weighted imaging^55,56^ and resting state functional connectivity^57^ in human subjects as well as in non-human primates using tracer techniques^58^. Several separate regions of the vestibular cortex are located within this Sylvian network, including the posterior insular cortex area (PIC), a region critical to the integration of visual and vestibular cues (for human subjects^59,60^; for non-human primates the region is referred to as VPS^58,61^). The information flow within this Sylvian network is not fully understood yet. Current theories assume that vestibular and visual cues about self motion are combined within PIVC and PIC and are then further processed to the temporoparietal junction (TPJ), a larger cortical region located at the junction of the temporal and parietal cortices, where visual-vestibular signals are integrated into a representation of the self in space^54^. The results of the current analysis support this view by providing first evidence for a potentially causal relationship with the supramarginal gyrus, which is part of the TPJ. Further functional connectivity between PIVC was observed with the visual cortex. This result is interesting, since several studies have shown inhibitory interactions between the visual system and PIVC^57,62–64^, such that PIVC is inhibited when visual cues are processed attentively and vice versa. As shown by magnetic resonance spectroscopy, this inhibition of PIVC is reflected by a decrease of excitatory neurotransmitter (glutamate and its precursor glutamine) within PIVC, concomitant with an increase in negative BOLD signal in PIVC^65^. These inhibitory interactions are assumed to be modulated in magnitude by attention networks located in the visual and parietal cortices^57^.

### Model generalization

Often one problem is the availability of a sufficient amount of data, in order to fully train and take advantage of machine learning models with large parameter spaces. Especially in MRI studies it is usually time-consuming and costly to acquire such large data sets. To account for this limitation, the concept of transfer learning was proposed in machine learning^32^. The basic idea behind transfer learning is that if only sparse data are available to learn a certain task, one can pretrain the model on a large-scale dataset of a similar task. In a next step, the feature representations learned on the large database can be used as an initialization for learning the desired target task. The goal is to transfer knowledge of one source domain to a target domain, by re-using the pretrained models weights. If the feature representation of the source domain is diverse enough, this can improve the model performance in comparison to starting the training without any prior knowledge, e.g. relying on a random initialization of the model weights^32^.

To investigate if transfer learning can also be suitable for our application, we studied the capabilities of the DCRNN to generalize across different datasets. Therefore we pretrained the DCRNN using the data provided by the HCP^33^, as described in the methods subsection ‘HCP data’. The model was pretrained for 70 epochs on in total 100 resting-state fMRI sessions, including the anatomical connectivity as reconstructed from DTI. Next we used a dataset acquired with a *Siemens Magnetom Prisma 3T* at the University of Regensburg (UR), where 10 different subjects participated in a resting-state fMRI session, including a DTI session, to acquire the corresponding structural imaging data. Each resting-state session of the UR dataset had a duration of 7.3 min, whereby 600 fMRI images were collected per scan. The acquisition parameters and the preprocessing involved are outlined in more detail in the subsection ‘UR data’. In analogy to the HCP dataset, the UR data was further processed by windowing the average BOLD signals in the regions defined by Glasser et al.^39^, thereby obtaining windows with an input and output length of $T_p=T_f=30$ timepoints. The first $80\%$ of these samples were used as a training data set, the subsequent $10\%$ for validation and the final $10\%$ for testing. We fine tuned the DCRNN, pretrained on the HCP data, by training it for 70 more epochs on the UR dataset, and initialized the second training with a lower learning rate of 0.001. This pretrained model was compared to the DCRNN, only trained on the UR dataset, and with weight parameters initialized randomly with Xavier/Glorot initialization^66^.

**Figure 5:**
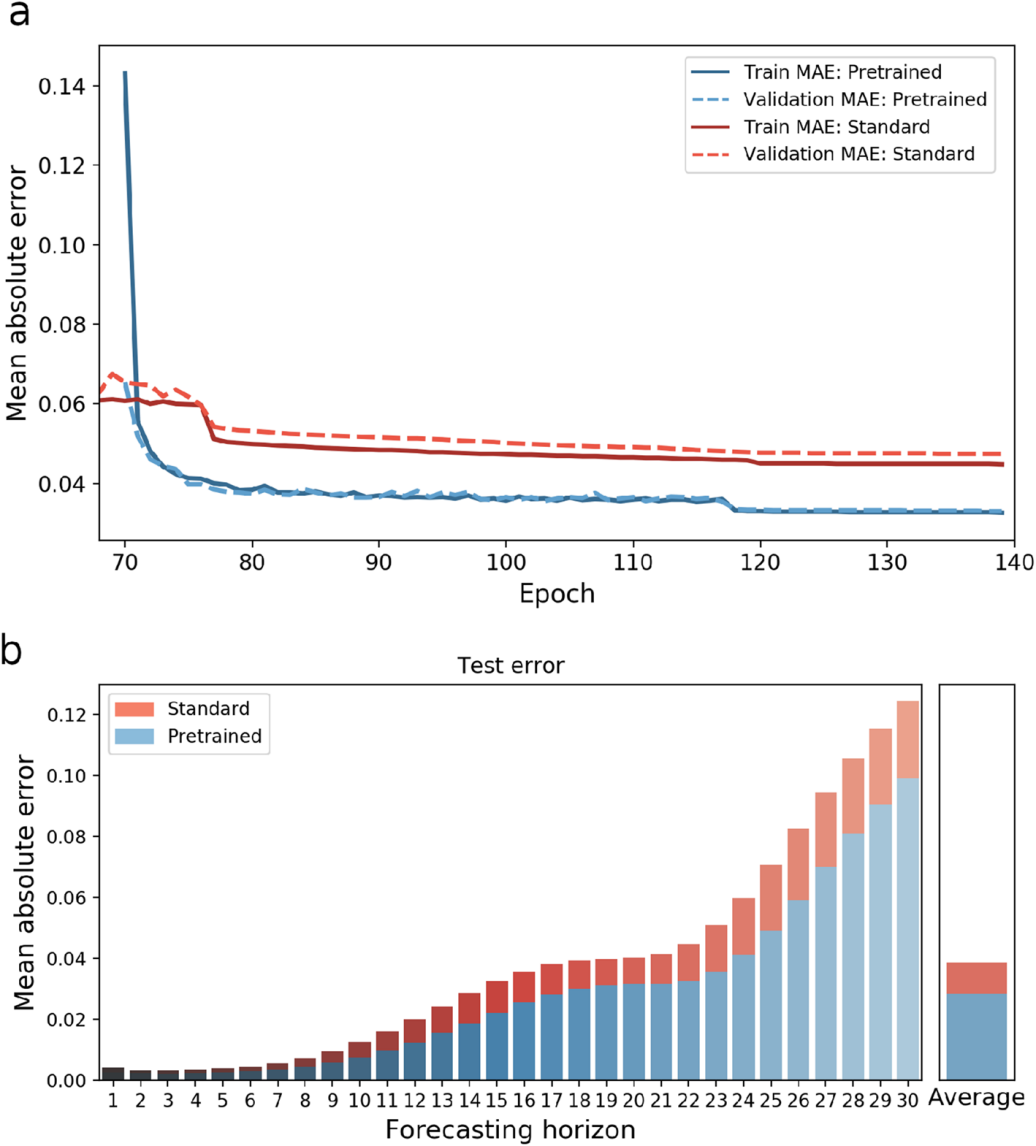
This figure illustrates the performance difference between standard training and encompassing transfer learning. Figure (**a**) shows the validation and training MAE during learning from epoch 70 onwards, and the errors with and without pretraining are depicted in blue and red respectively. The MAE values were computed as the average over all subjects, sessions, brain regions and test samples. At the very beginning of fine tuning, the error of the pretrained model is relatively high, but decreases after the model adapts to the UR dataset. In figure (**b**) the final test MAE of both models is shown in dependence on the forecasting horizon.

The comparison between relying on standard training, and utilizing transfer learning is illustrated in Fig. 5. Figure 5a shows the training and validation error during learning when starting with a random initialization of the weights in red. This model was trained in total for 140 epochs on the UR dataset only. In blue the training and validation error is depicted of the model, initially pretrained on the HCP dataset for 70 epochs, and fine tuned on the UR dataset for the subsequent 70 epochs. Figure 5a illustrates that at onset, the training error on the UR data is relatively high, but as the pretrained model adapts to the new dataset the MAE becomes considerably smaller than without pretraining. In Fig. 5b the test MAE in dependence of the prediction horizon is depicted. In total 540 test samples from 10 different subjects were used for the evaluation. The average test error could be reduced by $27 \%$ from 0.0388 to 0.0284 by encompassing transfer learning. Accordingly, the model performance on the small UR dataset, containing 10 sessions a 7.3 min becomes comparable to the performance on the large HCP dataset with 100 sessions a 14.4 min with a $MAE=0.0279$. Finally, to evaluate the significance across subjects, the test MAE with and without pre-training the model was computed for each of the 10 subjects. A paired t-test was applied and the difference was significant with $p \le 0.0001$.

## Discussion

We introduced a multi-modal framework for inferring causal relations in brain networks, based on a graph neural network architecture, uniting structural and functional information observed with DTI and fMRI. First this model provides a data-driven perspective on a fundamental question in neuroscience, namely how the function of the brain is related to its structure. Moreover, by modeling dynamic interactions on the structural anatomical substrate, this framework accounts for non-linear spatio-temporal dependencies between segregated brain regions, allowing us to reconstruct a multi-modal measure of causal influence strength.

First, we evaluated the performance of the DCRNN by studying its capabilities to reproduce empirically observed neural activity patterns, and compared it to a VAR model, like that typically used for the analysis of brain connectivity with Granger causality^21,46^. We showed that the DCRNN can also capture temporal long-term dependencies in fMRI data, enabling it to make accurate predictions up to $30 \, TRs$ ($\approx 20$ s) in the 0.04–0.07 Hz frequency range, which could reduced the overall test MAE considerably in comparison to a linear VAR. Note that results in subsection ‘Model performance’ demonstrate, that despite its simplicity, a VAR can make quite reliable predictions within the first $10 \, TRs$. Also its linearity allows for various possibilities for statistical inference of causal relations between different time courses, making it a feasible and fast tool for the estimation of Granger-causal connectivity^24^. But in the future it could be of interest to also consider non-linear and long-term relationships in neuroimaging data, in order to get a more complete picture of functional interactions between areas in the brain. The improved accuracy of the DCRNN reveals that it can better learn inherent characteristics of brain dynamics, and might therefore be more able to characterize causal relations than simple linear models. We further reproduced the analysis on a different dataset in Supplementary III, which could reveal that especially on small datasets it is beneficial to model transitions in brain networks with localized graph filters^18^. With this technique the predictions of the DCRNN remain stable also on large brain networks, even when only sparse data are available to fit this model to complex network structures. We additionally verified the results by employing a more liberal bandpass filter with cutoff frequencies 0.02–0.09 Hz in Supplementary IV. By including more frequency components, the BOLD signal becomes more complex and is accordingly harder to predict. The same analysis has been carried out relying on a volumetric brain atlas^49^, and using an alternative tractography method to reconstruct the structural connectivity in Supplementary V. In all cases the difference between the VAR and DCRNN in the prediction performance is apparent, especially for larger horizons. Also the DCRNN does not require stationarity of time series data, therefore avoiding potentially distorting pre-pocessing steps in order to achieve the latter. Another aspect that improves the plausibility of the estimated causal relations between brain regions is the integration of structural information into the graph neural network model. As the propagation of neural signals is physically constrained by the layout of white matter connections, propagating information via graph convolutions along anatomically connected regions is in agreement with prior knowledge about the anatomy of the brain.

The impact of this structural modeling was further investigated in the subsection ‘Impact of spatial modeling’. In the DCRNN the propagation of information is realized as a stationary diffusion process in the notion of a diffusion convolutions (DCs)^19^. Results show that diffusion steps of order $K=1$ already contribute most to the improvement of prediction accuracy, while higher order terms of $K=2,3$ only have a nominal further impact on the performance. The influence of structural modeling on the predictive performance provides additional insight into the general structure–function relation in the brain, by pointing out, how much additional information about the functional activity in a certain region can be gained from the inclusion of structurally connected regions. By including filters up to order $K=3$, the predictions could be improved by $27\%$ in comparison to when information from anatomically connected regions has been neglected. Note that for each time step *t* the DCRNN already applies multiple DCs to the multi-variate time series data, thereby inherently capturing the influence of higher order structural connections. Therefore low orders of diffusion walks $K \le 3$ seem to be already sufficient to account for indirect transitions. A good trade-off between computational load and model accuracy could be achieved with a maximum walk order of $K=2$, as the computational complexity linearly increases with *K*. Learning localized filters characterized by polynomial coefficients $\theta _k$ renders it possible to efficiently analyze large scale networks^18^, which allowed us to conduct an analysis with $N=360$ regions simultaneously on a single GPU. So unlike classical DCM, this model can also be applied to study interactions across the whole brain, making it suitable for an exploratory analysis.

The results demonstrated that propagating information across anatomical connections improves the model accuracy, pointing towards functional dependencies between different brain regions. In the spirit of explainable artificial intelligence (XAI), we proposed a method to reconstruct such dependencies, which the DCRNN has learned from the data in subsection ‘Causal connectivity’. Inducing perturbations in the model’s input space allowed us to study how the activity in a certain region influences other regions. This influence would quantify the importance of temporal information on the activity in a certain ROI for predicting the activity in other ROIs. Following the philosophy of Granger causality, this indicates a causal dependency between ROIs, thereby providing a measure of directed influence among each other. This kind of relation is referred to as *directed functional connectivity* or *causal connectivity*, as such information theoretic measures are dependent on causal mechanisms, but are not necessarily identical with them^67,68^, which distinguishes them from explicit model-based approaches like DCM for *effective brain connectivity*^10^. For our approach we used the more general notion of *causal connectivity*, as we do not only incorporate functional data, but also structural information to describe such causal dependencies between different regions. To demonstrate an application of our proposed approach, we evaluated the influence of PIVC on other brain regions. The derived connectivity network indicated a causal relationship between PIVC with brain regions in the Sylvian fissure, the perisylvian cortex and the insula, but also with the visual cortex.

In a final step, we proposed an approach to improve the model performance on smaller data sets. We demonstrated that the concept of transfer learning^32^ finds also an application in our context of detecting intrinsic patterns in fMRI time-series and structural connectivity data. Features learned from the data of the HCP repository^33^ could be well transferred to a smaller dataset, acquired with a *Siemens Magnetom Prisma 3T*. This made it possible to achieve almost the same accuracy on a small dataset with 10 sessions (each 7.3 min in duration) as with a large dataset of 100 sessions (each 14.4 min in duration). The acquisition and preprocessing protocols of the two fMRI datasets were relatively comparable in our study, so in other cases with larger differences in the temporal resolutions of the data, downsampling one dataset could be necessary in order for the model to better learn transferable feature representations.

Note that by integrating the structural information into the model, the functional interactions learned by this model also depend on the predefined anatomical layout. Therefore the quality of DTI data is additionally relevant for the results, but it is known that DTI has problems to accurately reconstruct long-range white matter tracks^69^. In our study we incorporated data from young healthy subjects and we computed a group SC matrix for the whole subject cohort, in order to model neural transitions on the anatomical substrate. Such transitions were characterized by local graph filters, which are optimized specifically for the underlying structural layout used in the model. In case of subject cohorts with very different SC profiles, like in studies including diseased patients or when comparing younger and older subjects, such graph filters would not generalize across cohorts and therefore have to be learned for the SC of every group individually. Also fMRI comes with its limitations for studying neural interactions, as the sampling rate is considerably lower than the underlying neural responses, and the neural activity is only indirectly measured based on the observed hemodynamic response^46^. An interdependence between the temporal information of two brain areas therefore only provides an indication for a causal relationship, and it should not necessarily be assumed to be identical with the latter. So the interpretation of the results should consider the informative content of the neuroimaging data used in this model. In our study we investigated possible applications of the GNN model using two different MRI data sets and different approaches for white matter tractography and frequency filtering (as outlined in the Supplementary Information). But for future studies, alternative imaging modalities and preprocessing schemes, could also be interesting for studying the structure–function relationship under a different light, for example by employing a log-transformed SC matrix to modify the influence of long-range structural connections, or observing functional dynamics in higher temporal resolution with electroencephalography (EEG) or magnetoencephalography (MEG).

In conclusion we think that GNN architectures can provide an interesting novel approach to combine complex non-linear temporal and spatial patterns as observed in fMRI and DTI data. Currently GNNs already show very promising applications for classification tasks in MRI based on brain connectivity networks^70–73^. In our study we showed that they can be also suitable to characterize the non-Euclidean spatial relationship of segregated brain regions when analyzing dynamic functional interactions on the structural network. Beyond the investigation of causal relations, this data-driven approach to brain dynamics could also be of interest for other applications. While many current approaches dealing with the structure–function relationship in the human brain focus on inferring the overall functional coherence patterns from their SC^6,26,28,29,31,74^, this framework allows us to directly relate temporally resolved activity profiles to their anatomical substrate. Further this whole-brain model could be interesting for clinical research, by studying dynamics in the diseased brain and observing how functional interactions between different areas might be affected. This multi-modal brain model could also be used to simulate the impact of a structural lesion to investigate the effects on the brain functions^75^. For detecting functional dependencies among different brain areas, we studied the signal changes in all other areas of the network, caused by a perturbation in a certain target area of interest. Alternative ways to look at such dependencies among the models input variables could be provided by approaches, like sensitivity analysis^76^ or layer-wise relevance propagation^77^, what might be of interest for future investigations in this area.

## Methods

### DCRNN

In the context of neuroimaging, neural activity patterns can be interpreted as a graph structured spatio-temporal signal distribution. The nodes in this graph represent ROIs in a human brain, while the edges reflect the connection strengths between these ROIs in the anatomical neuronal network, which forms a structural scaffold for the flow of information. This connection strength is given by the axonal connection strength as determined from DTI measurements. The activity dynamics on such networks can be modeled by a random walk on a graph, where a diffusion convolution operation is invoked to capture the spatial dependencies^18,19^. A diffusion–convolution recurrent neural network (DCRNN) is designed to integrate diffusion convolution, a sequence-to-sequence architecture and a scheduled sampling technique^19^. The model, as it is applied in the current study, is described in detail below.

When considering voxel time series of brain activity maps, we collect all data into a data matrix ${\mathbf {X}} = ({\mathbf {x}}(1) \ldots {\mathbf {x}}(T)),$ with ${\mathbf {x}}(t) \in {\mathbb {R}}^N$. Given *N* ROIs, taken from a brain atlas and each represented by a meta-voxel, and considering *T* time points for each meta-voxel time series, which represents the activation time course of one of the ROIs, then we have

3 $$\begin{aligned} {\mathbf {X}} = \left( \begin{array}{ccc} x_{11} &{} \cdots &{} x_{1T} \\ \vdots &{} x_{nt} &{} \vdots \\ x_{N1} &{} \cdots &{} x_{NT} \end{array} \right) \end{aligned}$$

Note that the columns ${\mathbf {x}}(t) \in {\mathbb {R}}^N$ of the data matrix describe the activation of all ROIs at any given time point $1 \le t \le T$, while its rows $\tilde{{\mathbf {x}}}_n(t), \; t=1, \ldots ,T$ represent the meta-voxel time course of every ROI $1 \le n \le N$.

Now consider a network of ROIs (brain areas, neuron pools) as an *undirected graph* $\mathcal {G} = ({\mathcal {V}},\mathcal {E},{{\mathbf {A}}}_w)$, where ${\mathcal {V}}, |{\mathcal {V}}| = N$ denotes a set of vertices (nodes), $\mathcal {E}$ represents a set of edges and ${{\mathbf {A}}}_w \in {\mathbb {R}}^{N \times N}$ is a *weighted adjacency matrix*. The latter represents the structural connectivity of the nodes, i.e. the ROIs on the neuronal network, which are adjacent to each other and connected by an edge. Such undirected graphs can be deduced from diffusion tensor imaging (DTI) data, which also provide the edge weights $w_{nn'}$. The latter reflect the anatomical connection strengths between the vertices. Note that DTI alone cannot determine the direction of information flow, what makes it necessary to incorporate functional imaging data.

The flow of activity observed on $\mathcal {G}$ is expressed as a time-dependent graph signal ${\mathbf {x}}(t) \in {\mathbb {R}}^{N}$. It represents the feature of each ROI, which here is the BOLD signal amplitude. Forecasting the flow of activity on $\mathcal {G}$ amounts to learning a function *h*(...) that maps $T_p$ past graph signals to future $T_f$ graph signals:

4 $$\begin{aligned} \left[ {\mathbf {x}}\left( t-T_p+1\right) , \ldots , {\mathbf {x}}(t); \mathcal {G}\right] \xrightarrow {h(...)} \left[ {\mathbf {x}}(t+1), \ldots , {\mathbf {x}}\left( t+T_f\right) \right] . \end{aligned}$$

#### Spatial dependencies

Information flow on $\mathcal {G}$ is considered a stochastic random walk process modeled by

- a re-start probability $\alpha \in [0,1]$
- a state transition matrix ${\mathbf {T}} = {\mathbf {D}}^{-1} {{\mathbf {A}}}_w = \left( \hat{{\mathbf {w}}}_1 \ldots \hat{{\mathbf {w}}}_N \right)$

Here we have with ${\mathbf {w}} \in {\mathbb {R}}^N$ and $\hat{{\mathbf {w}}}_n = \left( \hat{w}_{1n} \ldots \hat{w}_{Nn} \right) ^T \; \forall \; n =1, \ldots ,N$

5 $$\begin{aligned} {\mathbf {D}}= diag\left( {\mathbf {A}}_w {\mathbf {1}}\right) \end{aligned}$$

where the $\hat{w} = w_{nn'} / \sum _{n'} w_{nn'}$ denote normalized edge strengths. Here state transitions are modeled as a diffusion process on a graph. Note that because the DTI cannot obtain directed graphs, its diffusion matrix is symmetric, i.e. ${\mathbf {T}} = {\mathbf {T}}^T$. Thus an eigen-decomposition exists according to

6 $$\begin{aligned} {\mathbf {T}} = {\mathbf {U}}\varvec{\Lambda }{\mathbf {U}}^T. \end{aligned}$$

Further the state transition matrix ${\mathbf {T}}$ is proportional to a normalized graph Laplacian

7 $$\begin{aligned} {\mathbf {L}}_{rw} = {{\mathbf {I}}} - {\mathbf {T}} = {\mathbf {U}} \left( {\mathbf {I}} - \varvec{\Lambda } \right) {\mathbf {U}}^T \end{aligned}$$

representing a random walk on the graph. Now consider the set of eigenvectors ${\mathbf {U}}$ of the diffusion Laplacian matrix as a set of basis vectors. Then the graph signal ${\mathbf {x}}_t \in {\mathbb {R}}^N$ can be transformed to the conjugate domain and vice versa, hence we have^78^

8 $$\begin{aligned} {\mathbf {x}}_{\omega }= {\mathbf {U}}^T {\mathbf {x}}_{t} \end{aligned}$$

9 $$\begin{aligned} {\mathbf {x}}_{t}= {\mathbf {U}} {\mathbf {x}}_{\omega }. \end{aligned}$$

Finally invoking the convolution theorem, the *graph convolution operator* $*_G$ can be defined as

10 $$\begin{aligned} {\mathbf {y}}_{t} = {\mathbf {x}}_t *_G {\mathbf {f}}_{\theta } = {\mathbf {U}} \left( \left( {\mathbf {U}}^T {\mathbf {f}}_{\theta }\right) \odot \left( {\mathbf {U}}^T {\mathbf {x}}_t\right) \right) = {\mathbf {U}} \left( \varvec{\theta }_{\omega } \odot {\mathbf {x}}_{\omega } \right) , \end{aligned}$$

where ${\mathbf {f}}_{\theta }$ denotes a filter parametrized by $\theta$ and $\odot$ denotes the Hadamar product in the conjugate domain. The transformed vector ${\mathbf {U}}^T {\mathbf {f}}_{\theta } \equiv \varvec{\theta }_{\omega } = (\theta _1(\omega ), \ldots ,\theta _N(\omega ))^T$ summarizes the filter parameters $\theta _n, n = 1, \ldots , N$ into a parameter vector in the conjugate frequency domain. If it is replaced by a diagonal feature matrix, i.e. $\varvec{\theta }_{\omega } \rightarrow \varvec{\Theta }_{\omega } = {{\text{diag}}}(\theta _1(\omega ) \ldots \theta _N(\omega ))$, it represents a convolution kernel. Thus we have for the output signal

11 $$\begin{aligned} {\mathbf {y}}_t = {\mathbf {U}} \varvec{\Theta }_{\omega }{\mathbf {x}}_{\omega } = {\mathbf {U}} \varvec{\Theta }_{\omega } {\mathbf {U}}^T {\mathbf {x}}_t. \end{aligned}$$

Now expanding the filter kernel $\varvec{\Theta }_{\omega }$ into a power series with respect to the eigenvalue matrix $\varvec{\Lambda }$ of the transition matrix ${\mathbf {T}}$, unfolding the bi-quadratic form into a sum of rank one outer product forms $\theta _n {\mathbf {U}}{\mathbf {U}}^T , n=1, \ldots , N$, which can be considered elementary filter kernel, and finally keeping only terms up to order *K*, we obtain

12 $$\begin{aligned} {\mathbf {y}}_t & = {} {\mathbf {U}} \left[ \left( \sum _{k=0}^{K} \theta _k(\omega ) \varvec{\Lambda }^k \right) {\mathbf {U}}^T {\mathbf {x}}_t \right] \nonumber \\ & = {} \sum _{k=0}^{K} \theta _k(\omega ) {\mathbf {T}}^k {\mathbf {x}}_t. \end{aligned}$$

Note that this diffusion convolution operation includes the inverse diffusion process, represented by the transpose state transition matrix ${\mathbf {T}}^T$ as well, since DTI can only yield undirected graphs. Thus, as has been shown by^19^, diffusion convolution is intimately related to spectral graph convolution (SGC)^18^. More precisely, GDC is equivalent to SGC up to a similarity transformation^19^.

Considering a CNN architecture and using the diffusion convolution operation, the output of each of the $q \in \{1,\ldots ,Q\}$ diffusion convolution layers (DCL) is then given as follows:

13 $$\begin{aligned} {\mathbf {h}}_{q,t} = \sigma \left( {\mathbf {y}}_{q,t} \right) = \sigma \left( \sum _{k=0}^{K} \theta _{k,q} {\mathbf {T}}^k {\mathbf {x}}_t \right) . \end{aligned}$$

Hereby ${\mathbf {x}}_t \in {\mathbb {R}}^{N}$ denotes the input at time *t*, ${\mathbf {h}}_{q,t} \in {\mathbb {R}}^{N}$ the corresponding output of the *q*th convolution layer, *Q* the number of filters employed, $\sigma (...)$ any suitable activation function, and $\theta _{q,k} \in {\mathbb {R}}^{K+1}$ parameterizes the *q*-th convolutional kernel of order *k*. The DCL learns to represent graph structured data and can be trained with gradient descent based optimization techniques.

Note that this random walk on a graph represents a Markov process. At the limit $K \rightarrow \infty$ it converges to a stationary distribution ${\mathbf {P}} \in {\mathbb {R}}^{N \times N}$, which for finite $K < \infty$ can be approximated by^79^

14 $$\begin{aligned} {\mathbf {P}} = \sum _{k=0}^{K} \alpha (1 - \alpha )^k {\mathbf {T}}^k. \end{aligned}$$

The *i*-th row ${\mathbf {P}}_{i,*}$ of this matrix represents the likelihood of diffusion starting from ROI $v_i \in {\mathcal {V}}$, hence the proximity of any other ROI $v_j \in {\mathcal {V}}$ with respect to ROI $v_i$.

#### Temporal dependencies

**Figure 6:**
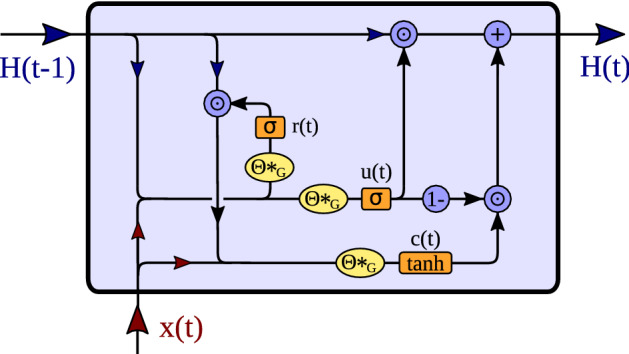
Overview of the processing steps of the DCGRU cell. The input ${\mathbf {x}}(t)$, as well as the previous hidden state ${\mathbf {H}}(t-1)$ are concatenated and passed to the reset gate ${\mathbf {r}}(t)$, as well as to the update gate ${\mathbf {u}}(t)$. The reset gate ${\mathbf {r}}(t)$ controls the proportion of ${\mathbf {H}}(t-1)$ which enters ${\mathbf {c}}(t)$, together with input ${\mathbf {x}}(t)$. Then the hidden state ${\mathbf {H}}(t-1)$ is updated by ${\mathbf {c}}(t)$, whereby the amount of new information is controlled by ${\mathbf {u}}(t)$.

Given the graph convolution operation, temporal dynamics on the graph can be modeled using gated recurrent units (GRU)^36^. The trick is to replace the matrix multiplications in GRU by diffusion convolutions $*_G$, as derived in Eq. (12). This leads to the diffusion convolutional gated recurrent unit (DCGRU)^19^

15 $$\begin{aligned} {\mathbf {r}}(t)= \sigma \left( \varvec{\Theta }_r *_G \left[ {\mathbf {x}}(t), {\mathbf {H}}(t-1)\right] + {\mathbf {b}}_r \right) \end{aligned}$$

16 $$\begin{aligned} {\mathbf {u}}(t)= \sigma \left( \varvec{\Theta }_u *_G \left[ {\mathbf {x}}(t), {\mathbf {H}}(t-1) \right] + {\mathbf {b}}_u \right) \end{aligned}$$

17 $$\begin{aligned} {\mathbf {c}}(t)= \tanh \left( \varvec{\Theta }_c *_G \left[ {\mathbf {x}}(t), ({\mathbf {r}}(t) \odot {\mathbf {H}}(t-1)) \right] + {\mathbf {b}}_c \right) \end{aligned}$$

18 $$\begin{aligned} {\mathbf {H}}(t)= {\mathbf {u}}(t) \odot {\mathbf {H}}(t-1) + \left( 1 - {\mathbf {u}}(t)\right) \odot {\mathbf {c}}(t), \end{aligned}$$

where ${\mathbf {x}}(t), {\mathbf {H}}(t)$ denote the input and output states of the GRU at time *t* and $[{\mathbf {x}}(t),{\mathbf {H}}(t-1)]$ denotes their concatenation. Also ${\mathbf {r}}(t), {\mathbf {u}}(t)$ represent reset and update gates at time *t*, and ${\mathbf {b}}_r, {\mathbf {b}}_u, {\mathbf {b}}_c$, respectively, denote bias terms. Furthermore, $\varvec{\Theta }_r, \varvec{\Theta }_u, \varvec{\Theta }_c$ denote the parameter sets of the corresponding filters. An illustration of a single DCGRU cell is provided in Fig. 6.

Similar to GRUs, also DCGRUs can be employed to build layers of recurrent neural networks, which can be trained by backpropagation through time (BPTT)^80,81^. If multiple step ahead forecasting is intended, a sequence-to-sequence architecture can be used. In this architecture, both the encoder and the decoder are composed of DCGRU layers forming a diffusion convolution recurrent neural net (DCRNN) (see Fig. 1). During training, a time series of past events is fed into the encoder and its final states form the input to the decoder. The latter then generates predictions, which can be compared to available ground truth observations. For later testing, such ground truth observations are replaced by predictions generated by the model itself. Given BOLD signal voxel activation time series, segments of an observed voxel time series are used to train a DCRNN to predict future activations.

#### Training the DCRNN

The network is trained by maximizing the likelihood of generating the target future time series using BPTT learning. Hence, DCRNN can capture spatio-temporal dependencies between time series. After the Bandpass filtering of the BOLD signal in each region, the data of each fMRI session was scaled between 0 and 1 before starting the training. The DCRNN^19^ was implemented using the *TensorFlow*^82^ library for machine learning, and computations were performed on an *Nvidia Quadro K6000* GPU, running on a desktop PC with an *Intel(R) Xeon(R) CPU E5-1620 v4* CPU under *Linux Debian 9*. Scheduled sampling^37^ is invoked during training to account for the fact that the distribution of input stimuli during testing might differ from the distribution of training stimuli. During scheduled sampling reference observations are fed to the model with probability $\epsilon _i$, while predictions released by the model are fed in with probability $1 - \epsilon _i$ at the *i*-th iteration. During supervised training, instances to be predicted are, of course, known. An inverse sigmoidal function determines the sampling probability decay:

19 $$\begin{aligned} \epsilon _i = {\frac{\tau }{1-exp(i/\tau )}}. \end{aligned}$$

It was found to be sufficient to train the model for 70 epochs, and the scheduled sampling parameter can be set to $\tau =5000$. As an objective function the mean absolute error (MAE) was used to describe the overall difference between true activity ${\mathbf {x}}(t)$ and predicted activity $\hat{{\mathbf {x}}}(t)$:

20 $$\begin{aligned} \mathbf {MAE}\left( {\mathbf {x}} , \hat{{\mathbf {x}}}\right) = \frac{1}{T_f} \sum _{t=1}^{T_f} |{\mathbf {x}}(t) - \hat{{\mathbf {x}}}(t)|. \end{aligned}$$

**Figure 7:**
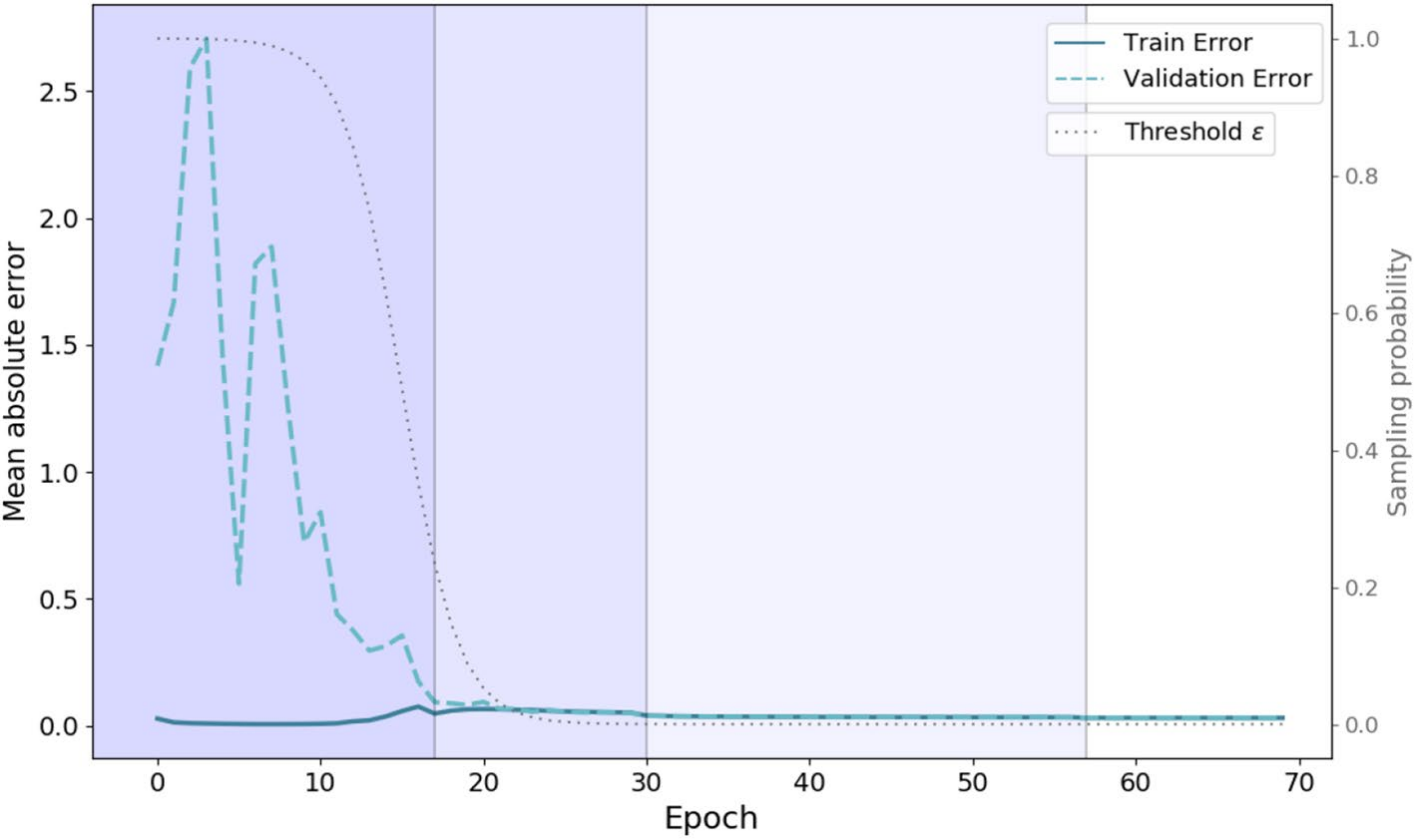
Illustration of the model performance during training. The figure shows the MAE during the training (solid blue line) and validation data set (dashed light blue line) in dependence of the number of epochs. The gray line illustrates the scheduled sampling probability $\epsilon _i$ over time. Vertical lines indicate when the learning rate was lowered by a factor of 0.1. In the first few epochs the training error, due to the high schedule sampling probability $\epsilon _i$, is already quite low. During testing and validation the inputs for the decoder are always the models own predictions, what reflects the large discrepancy between training and validation error within the first epochs. When the sampling probability is subsequently decreased, the model also learns to successfully make long term forecasting.

For this optimization problem, the ADAM algorithm^83^ was employed. The samples in the training data set were randomly permuted and the gradient was derived from mini-batches of 32 samples. To achieve a good convergence and to avoid too strongly growing gradients, it was found useful to use an annealing learning rate, initialized with $\eta = 0.1$. The learning rate was decreased by a factor of 0.1 at epochs 20, 40 and 60, or if the validation error did not improve for more than 10 epochs. Before lowering the learning rate, the weights with lowest validation error were restored, in order to avoid getting stuck in local optima. The encoder and decoder of the sequence-to-sequence architecture consist to two diffusion convolution GRU layers each, and the hidden state size is set to $Q=64$. The training performance is illustrated in Fig. 7. Due to the curriculum learning strategy, within the first few epochs the probability of the decoder receiving a true label is very high, like that illustrated by the gray line in Fig. 7. Therefore at onset of the training, the model has to make only correct short term predictions and the training MAE is already relatively small, indicated by the solid dark blue line. For validation and testing, the decoder always receives its own previous prediction. The validation MAE is illustrated with the dashed light blue line, and it can be seen that the model gradually learns to make also accurate long term predictions. Finally, for the evaluations the whole population dataset was scaled to unit variance and zero mean.

### Autoregressive models

As Granger causality^21^ is typically based on linear vector autoregressive (VAR) models for stochastic time series data, we evaluated a VAR as one baseline method. The idea of an autoregressive process (AR) is that a time series *x*(*t*) can be described by a linear function of the first $T_p$ of its lagged values^84^

21 $$\begin{aligned} x(t) = \beta + \alpha _1 x(t-1) + \alpha _2 x(t-2) + \dots + \alpha _p x\left( t-T_p\right) + u(t) \end{aligned}$$

with coefficients $\alpha _1 \dots \alpha _p$, intercept $\beta$ and an error term *u*(*t*). This expression can be extended to a multivariate VAR model with *N* time series ${\mathbf {x}}(t) = [x_1(t), \dots , x_N(t)]$ as^84^

22 $$\begin{aligned} {\mathbf {x}}(t) = {\mathbf {b}} + {\mathbf {A}}_1 {\mathbf {x}}(t-1) + {\mathbf {A}}_2 {\mathbf {x}}(t-2) + \dots + {\mathbf {A}}_p {\mathbf {x}}\left( t-T_p\right) + {\mathbf {u}}(t), \end{aligned}$$

where coefficients are stored in matrices ${\mathbf {A}} \in {\mathbb {R}}^{N \times N}$, and intercepts and errors are described by vectors ${\mathbf {b}} \in {\mathbb {R}}^{N}$ and ${\mathbf {u}}(t) \in {\mathbb {R}}^{N}$. In the context of this study, time series ${\mathbf {x}}(t)$ reflect the BOLD signal of *N* brain regions, measured at different times *t*.

For the estimation of coefficients ${\mathbf {A}}$ and intercepts ${\mathbf {b}}$ various methods exist^24^, and in this study we rely on two different strategies. The first is based on a typical ordinary least squares (OLS) estimation^24,47^ on individual subject sessions, implemented in the *statsmodel* python package^85^. The first $80 \%$ of each fMRI session were used to fit the model to the data, and the subsequent $10 \%$ were used for validation and the last $10 \%$ were employed as a test set. To check for stationarity of the analyzed time series an augmented Dickey–Fuller test for unit roots was performed^47,86^, with a p-value of $p<0.01$.

Additionally, in order to render the comparison to the DCRNN more accurate, we implemented a gradient descend based optimization for a VAR model in *TensorFlow*^82^, to verify that the differences in predictive performance can be related to the models, and not solely to the optimization strategies. In analogy to the DCRNN training, input-output samples of neural activities were generated from the data like described in subsection ‘Data description’, which were used to minimize the MAE between the model’s prediction ${{\hat{\mathbf{x}}}}(t)$ and groundtruths ${\mathbf {x}}(t)$. The convergence could be optimized by employing stochastic gradient descent (SGD) optimization with a batch size of 1, using an annealing learning rate with a start value of $\eta = 0.005$. The VAR model was trained for 100 epochs, and the learning rate was reduced by a factor of 0.1 after epoch 70 and 90. A comparison of the error on the test set between the two different optimization strategies can be found in supplement VI.

Best performance could be achieved employing a SGD based optimization in combination with a lag order of $P=30$. Note that with such a high lag order, around $9.7\%$ of the $N=360$ time courses do not fulfill the stationarity criteria of the augmented Dickey–Fuller test anymore ($p>0.01$). Yet the prediction accuracy could still be improved by including lags up to $P=30$, like shown in supplement VI. As the objective criterion of the evaluation was to assess the capabilities of replicating empirically observed neural activity patterns, we chose the VAR model with best accuracy for comparison with the DCRNN in ‘Model performance’.

### Datasets

#### HCP data

The first data set used in this study is provided by the HCP data repository^33,87^. The S1200 release includes data from subjects which participated in four resting state fMRI sessions, lasting 14.4 min each and collecting 1200 volumes per session. Customized *Siemens Connectome Skyra* magnetic resonance imaging scanners with a field strength of $B_0 = 3$ T were employed for data acquisition, using multi-band (factor 8) acceleration^88–91^. The data was collected by gradient-echo echo-planar imaging (EPI) sequences with a repetition time $TR = 720$ ms and an echo time $TE = 31.1$ ms. The field of view was $FOV = 208\ {\text {mm}} \times 180\ {\text {mm}}$ and $N_s = 72$ slices with a thickness of $d_s = 2$ mm were obtained, containing voxels with a size of $2\ {{\text {mm}}} \times 2\ {{\text {mm}}} \times 2\ {{\text {mm}}}$. The preprocessed version, including motion-correction, structural preprocessing and ICA-FIX denoising was chosen^38,92–97^. Next a multi-model parcellation scheme was applied to divide the cortical gray matter hemisphere into 180 regions^39^, and the BOLD signal inside each region was averaged, to obtain the temporal activity evolution for each area. For our study we found it appropriate to apply global signal regression, firstly because it showed to effectively reduce movement artifacts in HCP datasets^98^. Also in this study of causal relations, the goal was to extract the additional information, which certain brain regions contain about the activity of other regions, whereby local interactions rather than global modulations were of interest for us. Those time courses were bandpass filtered, first performing the evaluations on a noise reduced narrowband in ‘Model performance’, employing a filter with cutoff frequencies 0.04–0.07 Hz^40–43^, and additionally implementing a more liberal bandpass filter with cutoff frequencies 0.02–0.09 Hz, as displayed in Supplementary IV.

Diffusion MRI data was collected in 6 runs, whereby approximately 90 directions were sampled during each run, employing three shells of $b=1000, 2000,$ and $3000 \ {\text {s}}/{{\text {mm}}}^2$, including 6 $b=0$ images^99^. A Spin-echo EPI sequence was employed with repetition time $TR = 5520\ {\text {ms}}$, echo time $TE = 89.5\ {\text {ms}}$, and multi band factor 3. In total $N_s = 111$ slices were obtained, with field of view $FOV = 210\ {{\text {mm}}} \times 180\ {{\text {mm}}}$ and an isotropic voxel size of $1.25\ {{\text {mm}}} \times 1.25\ {{\text {mm}}} \times 1.25\ {{\text {mm}}}$. The preprocessing included intensity normalization across runs, EPI distortion correction, eddy-current corrections, removing motion artifacts, and gradient non-linearity corrections^38,100–103^. To obtain the structural connectivity strengths between regions defined by Glasser et al.^39^, the *MRtrix3* software package was employed^35^. Briefly multi-shell multi-tissue constrained spherical deconvolution^45^ was used to obtain the response functions for fiber orientation distribution estimation^104,105^. Furher 10 million streamlines were created with anatomical constrained tractography^106^ and spherical-deconvolution informed filtering was applied^107^, reducing the number of streamlines to 1 million. To quantify the strength of the structural connections, the number of streamlines connecting two brain regions were computed, and normalized by the region volumes. A detailed description of the workflow can be found in: https://osf.io/fkyht/. The group structural connectome was computed as an average across the first 10 subjects, as the variance in the structural connectivity strength is relatively low across subjects^108^, while probabilistic tractography methods are relatively computationally demanding. For this dataset, including only young healthy subjects, the similarity of the SC across subjects was relatively high, and the correlation coefficient between SC values of different subjects was on average 0.91. But note that when comparing very different subject cohorts, like healthy and diseased subjects, the SC matrix should be computed for every studied group individually.

#### UR data

The second dataset was acquired with a *Siemens Magnetom Prisma* with field strength $B_0 = 3$ T at the University of Regensburg (UR). The data of 10 different subjects were used, whereby resting state fMRI data were collected during a scanning time of 7.3 min. All subjects provided written informed consent and the study was approved by the local ethics committee of the University of Regensburg. All methods were performed in accordance with the relevant guidelines and regulations. An EPI sequence was employed using multi-band (factor 8) acceleration, sampling 600 volumetric images per run with a repetition time of $TR= 730$ ms and an echo time of $TE=31$ ms. The field of view was $FOV = 208 \ {{\text {mm}}} \times 208 \ {{\text {mm}}}$ and $N_s = 72$ slices with thickness of $d_s = 2 \ {{\text {mm}}}$ were collected, containing voxels with a size of $2 \ {{\text {mm}}} \times 2 \ {{\text {mm}}} \times 2 \ {{\text {mm}}}$. For preprocessing the HCP pipeline (version 4.0.0) was employed, as described by Glasser et al.^38^. To achieve good correspondence between the two datasets, the further preprocessing was also performed as outlined in subsection ‘HCP data’. The fMRI time courses were averaged within each brain region of the multi-modal parcellation scheme^39^, and again global signal regression was applied. Finally those time courses were bandpass filtered within the noise reduced range of 0.04–0.07 Hz.

To reconstruct the anatomical connectivity, diffusion MRI data was collected in 4 runs, sampling approximately 90 directions, employing two shells with $b = 1500$ and $3000 \ {\text {s}}/{{\text {mm}}}^2$, and also including 7 $b=0$ images. The repetition time of the Spin-echo EPI sequence was $TR=3222$ ms with an echo time $TE=89.2$ ms, employing a multi-band (factor 4) acceleration. Overall $N_s=92$ slices were collected, with a field of view $FOV = 210 \ {{\text {mm}}} \times 210 \ {{\text {mm}}}$, containing voxels with a size of $1.5 \ {{\text {mm}}} \times 1.5 \ {{\text {mm}}} \times 1.5 \ {{\text {mm}}}$. Preprocessing of the diffusion MRI data was based on the HCP guidelines^38^, and finally the anatomical connectivity matrices were obtained like in the previous subsection ‘HCP data’, using constrained spherical deconvolution as provided in the *MRtrix* package^35^. The group structural connectivity was computed as an average over the 10 subjects.

## Supplementary Information

Supplementary Information.
