# Supplementary material for: A graph neural network framework for causal inference in brain networks
Source: Sci Rep. 2021 Apr 13;11:8061. doi: 10.1038/s41598-021-87411-8 (PMC8044149; doi:10.1038/s41598-021-87411-8)
Supplement: Supplementary file 1 — Supplementary Information. [file 41598_2021_87411_MOESM1_ESM.pdf]

A Graph Neural Network Framework for Causal Inference in Brain Networks

S. Wein[1,2], W. M. Malloni[2], A. M. Tomé[3], S. M. Frank[4], G.-I. Henze[2], S. Wüst[2], M. W. Greenlee[2], and E. W. Lang[1]

[1]CIML, Biophysics, University of Regensburg, Regensburg, Germany
[2]Experimental Psychology, University of Regensburg, Regensburg, Germany
[3]IEETA/DETI, Universidade de Aveiro, Aveiro, Portugal
[4]CLPS, Brown University, Providence, RI 02912, USA

Supplement I

In the section 'Model performance' the test data was used from subjects, from which in part also the training data was generated. To test how well the trained models generalize to a new subject cohort, we have generated a second test dataset, with in total 4 rs-fMRI sessions from 10 unseen subjects. The second test datasets contains therewith in total 45640 samples, and the results are illustrated in figure S1.

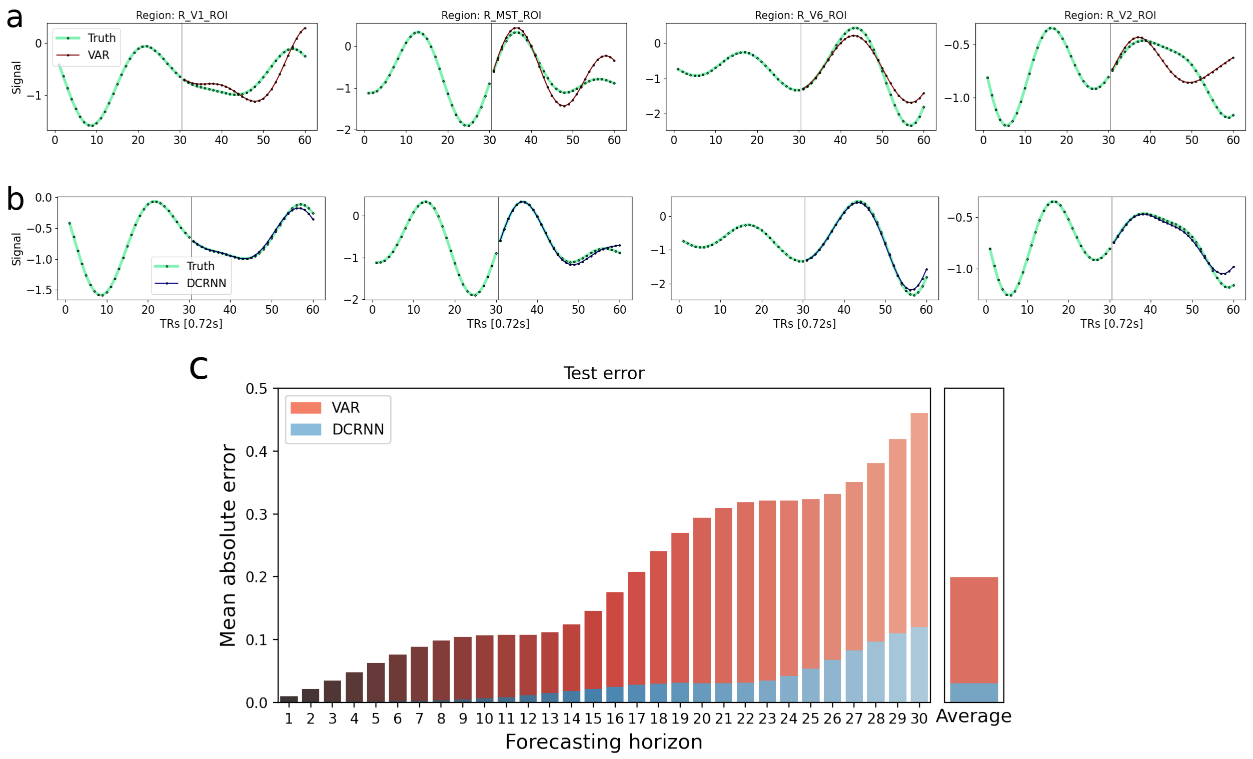

Figure S1: The figure illustrates the prediction accuracy of a VAR model (a) in comparison to the DCRNN (b) on test data from unseen subjects. The prediction error of the VAR model on this representative sample is with 0.199 marginally below average, while the error of the DCRNN is with 0.037 higher than its average. Below the average MAE over all samples in the test set is illustrated, in dependence of the forecasting horizon (c).

The test error of the DCRNN increased from 0.0279 to 0.0306 and for the VAR from 0.1786 to 0.1991 when predicting the BOLD signal of unseen subjects, but the performance difference between the DCRNN and VAR remained the same. To test the significance of this difference across subjects, the test MAE for each individual subject was computed. A paired t-test was applied, which was significant with a value of $p \leq 0.0001$.

In a next step we tested the impact of the size of the training data. Figure S2 (a) shows the test MAE in dependence on different number of fMRI sessions incorporated for training the DCRNN model. The training data was always generated by using the first 80% of each fMRI session, corresponding to 913 training samples per session. In order to employ the same test set for every training setup, the evaluation was performed on the test dataset of 10 new unseen subjects again. The results suggest that after using data from 25 sessions, the model is still able to improve, but the performance then saturates around roughly 50 sessions. Further in figure S2 (b) we studied the role of information in past neural activity for prediction future activity values. The goal of the model was to infer the subsequent $T_f = 30$ BOLD signal values. Figure S2 (b) illustrates that only $T_p = 10$ input values are not sufficient in order to make accurate long-term predictions, but the prediction error can be effectively reduced by incorporating more information from the past.

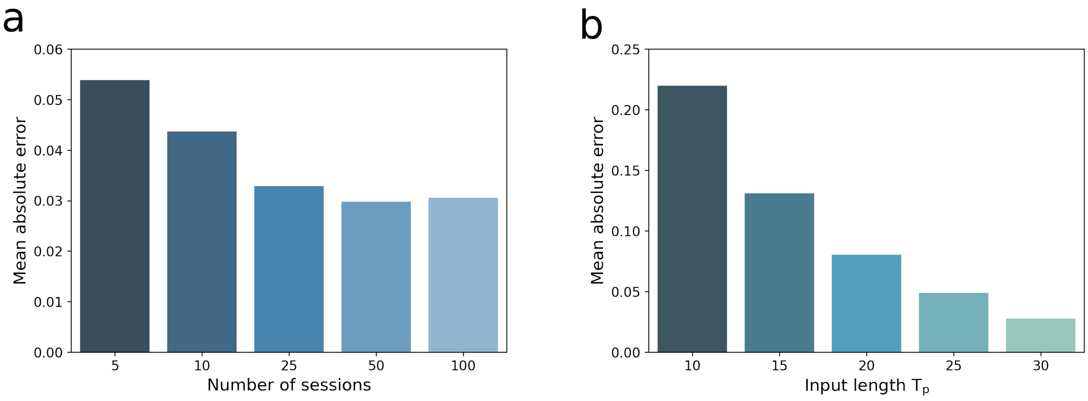

Figure S2: This figure illustrates the impact of the training data set size on the prediction MAE (a), as well as the the role of input timeseries length T_p on the model performance (b).

In a subsequent analysis we investigated the consistency of the prediction performance across subjects. The test MAE was computed for every individual subject and figure S3 (a) illustrates the variability of the prediction accuracy across those subjects. The diagram suggests that the performance of the DCRNN model can be consistently reproduced in a cohort of young healthy subjects. Further we visualized the characteristics of the test MAE in different brain regions, to investigate if there are areas where the BOLD signal could be predicted more or less accurately. For this purpose the average test MAE was computed for each of the 360 regions and the variability across the regions is illustrated in figure S3 (b). To study this variability in more detail, the error in different ROIs were projected onto the surface of the multi-modal parcellation atlas [3] and the resulting brain map is depicted in figure S3 (c). Overall the prediction error is relatively homogenous distributed across different regions and mainly in the posterior cingulate cortex a considerably larger error can be observed. This could point towards a higher variability of neural activity patterns in the posterior cingulate cortex, what might suggest that more complex neural computations occur in this brain region, while subjects mind wander and do not perform any particular task during the resting state scan [8].

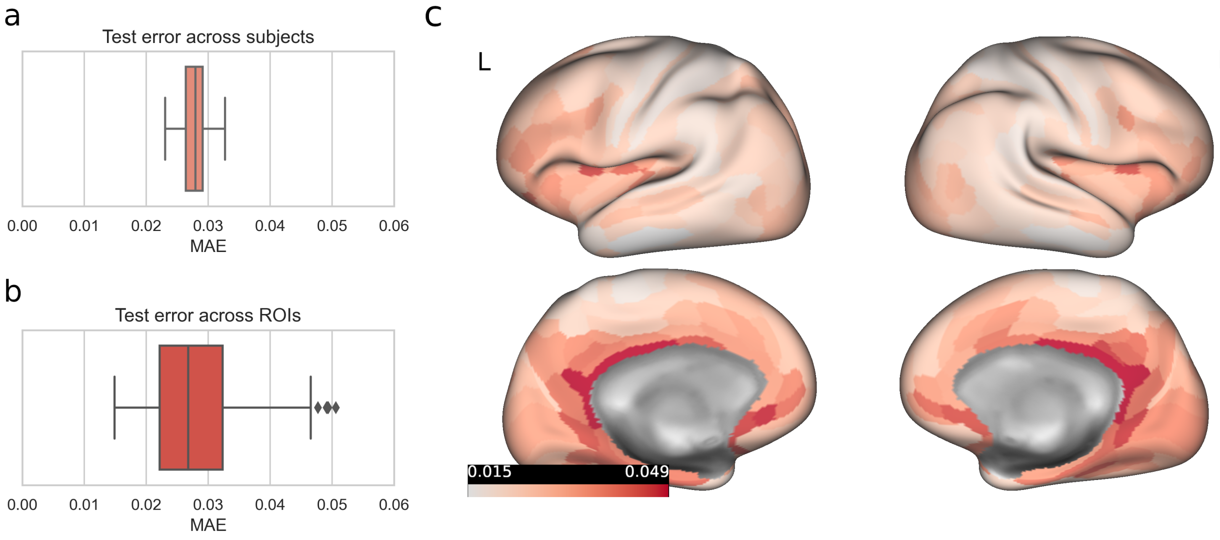

Figure S3: The figure illustrates the variability of the test error across different subjects and brain regions. The diagram in (a) shows the distribution of MAE values across subjects, while in (b) the MAE distribution across ROIs is visualized. Further in (c) we projected the error values on the 360 brain regions defined by Glasser et al. [3] in the left and right hemisphere. The test MAE values are encoded in red in the different areas. Overall the distribution of the prediction error is also relatively homogenous across ROIs and mainly in the posterior cingulate cortex a stronger prediction error can be observed. The figure was created with the Connectome Workbench software (version 1.4.2): https://www.humanconnectome.org/software/connectome-workbench.

Supplement II

In this section we further motivate the choice of neural network architecture for the DCRNN model by comparing it to different baseline models. The first model consists of simple gated recurrent unit (GRU) cells [2], modified to process graph structured signals, without employing a sequence to sequence learning strategy in an encoder-decoder architecture. Like described in 'Spatial dependencies' in the 'Methods' section (equations 15-18), the gating mechanisms of the GRU cells invoke diffusion convolutions (DCs) operations to model transitions on the graph structured signals. In addition to the GRUs we implemented a long-short term memory (LSTM) neural network [5], which incorporates DC operations in the following way:

$$\mathbf{f}(t) = \sigma\left(\mathbf{\Theta}_f *_G [\mathbf{x}(t), \mathbf{H}(t-1)] + \mathbf{b}_f\right) \tag{1}$$

$$\mathbf{i}(t) = \sigma\left(\mathbf{\Theta}_i *_G [\mathbf{x}(t), \mathbf{H}(t-1)] + \mathbf{b}_i\right) \tag{2}$$

$$\mathbf{o}(t) = \sigma\left(\mathbf{\Theta}_o *_G [\mathbf{x}(t), \mathbf{H}(t-1)] + \mathbf{b}_o\right) \tag{3}$$

$$\tilde{\mathbf{c}}(t) = \tanh\left(\mathbf{\Theta}_{\tilde{c}} *_G [\mathbf{x}(t), \mathbf{H}(t-1)] + \mathbf{b}_{\tilde{c}}\right) \tag{4}$$

$$\mathbf{C}(t) = \mathbf{f}(t) \odot \mathbf{C}(t-1) + \mathbf{i}(t) \odot \tilde{\mathbf{c}}(t) \tag{5}$$

$$\mathbf{H}(t) = \mathbf{o}(t) \odot \tanh(\mathbf{C}(t)) \tag{6}$$

with $\mathbf{x}(t), \mathbf{H}(t)$ denoting the input and output states of the LSTM at time t and $[\mathbf{x}(t), \mathbf{H}(t-1)]$ denotes their concatenation. Further $\mathbf{f}(t), \mathbf{i}(t)$ and $\mathbf{o}(t)$ represent the forget, input and output gates at time t, and $\mathbf{b}_f, \mathbf{b}_i, \mathbf{b}_o$, respectively, denote bias terms. The candidate state is represented by $\tilde{\mathbf{c}}$, with the corresponding bias $\mathbf{b}_{\tilde{c}}$. Furthermore, $\mathbf{\Theta}_f, \mathbf{\Theta}_i, \mathbf{\Theta}_o$ and $\mathbf{\Theta}_{\tilde{c}}$ denote the parameter sets of the graph filters.

We now compared the GRU and LSTM, modified to process graph structured signals, to the DCRNN and also to the VAR model. The hidden state sizes of the GRU and LSTM were set

to $Q = 64$, and the 2 concatenated cells build up the models respectively. The baseline models were then also trained on the HCP dataset like outlined in the section ‘Data description’ in the manuscript, and the comparison of the test MAEs is illustrated in figure S4. It can be seen that all models can make reasonable accurate predictions within the first 10 timesteps, but the difference then becomes clearly apparent in the long-term forecasting. Both, the GRU and LSTM models were not able to make robust inferences for larger prediction horizons, with an overall test $MAE = 0.3281$ and $MAE = 0.4846$ respectively, which exceeded the test error of the VAR model ($MAE = 0.1786$) and the DCRNN ($MAE = 0.0279$). Again a paired t-test was applied, to test the significance in the performance difference between DCRNN and GRU, as well as the DCRNN and LSTM architecture, which both were significant across subjects with a value of $p \leq 0.0001$. This points towards the advantage of using an encoder-decoder architecture [9], as implemented in the DCRNN, in order to fully capture long-term dependencies in the BOLD signal.

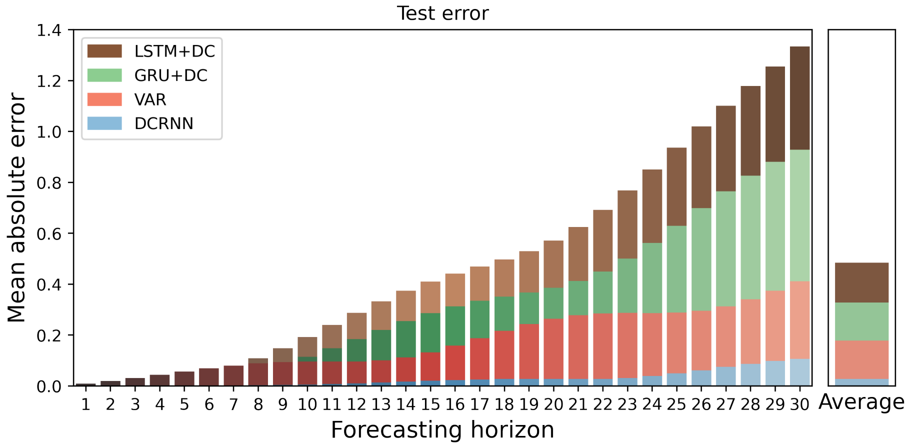

Figure S4: The figure shows a comparison between a LSTM and GRU model, extended for processing graph signals by invoking DC operations, with the VAR and DCRNN model. The test error is depicted in dependence of the forecasting horizon, and was computed as an average across subjects, sessions, ROIs and samples.

Supplement III

In this section we reproduce the comparison with the VAR model, using the dataset collected at the University of Regensurg (UR), which is described in more detail in the subsection ‘UR data’ in the manuscript. This dataset includes resting-state fMRI sessions from 10 different subjects, as well as their structural imaging data. Again the first 80% of the samples of each fMRI session were used to train the models, so the overall training data size is with 4330 samples considerably smaller than the training data size of the HCP dataset with in total 91300 samples. Considering a network with N nodes, the number of parameters in a VAR model scale with an order of N^2, so for a large network size N, the number of parameters would strongly exceed the number of available training samples. We therefore compared the models on only a subset of regions of the multi-modal parcellation atlas [3] at first, using the first $N = 40$ ROIs. The prediction accuracy of the two models for this network size is illustrated in more detail in figure S5 (a), (b) and (c). For a medium networksize of $N = 40$, the overall test error of the VAR model is with $MAE = 0.3165$ larger than the prediction error of the DCRNN with $MAE = 0.0665$. To test the significance in the performance difference across subjects, the overall MAE was computed for every individual subject. A paired t-test was applied which showed to be significant with a value of $p \leq 0.0001$.

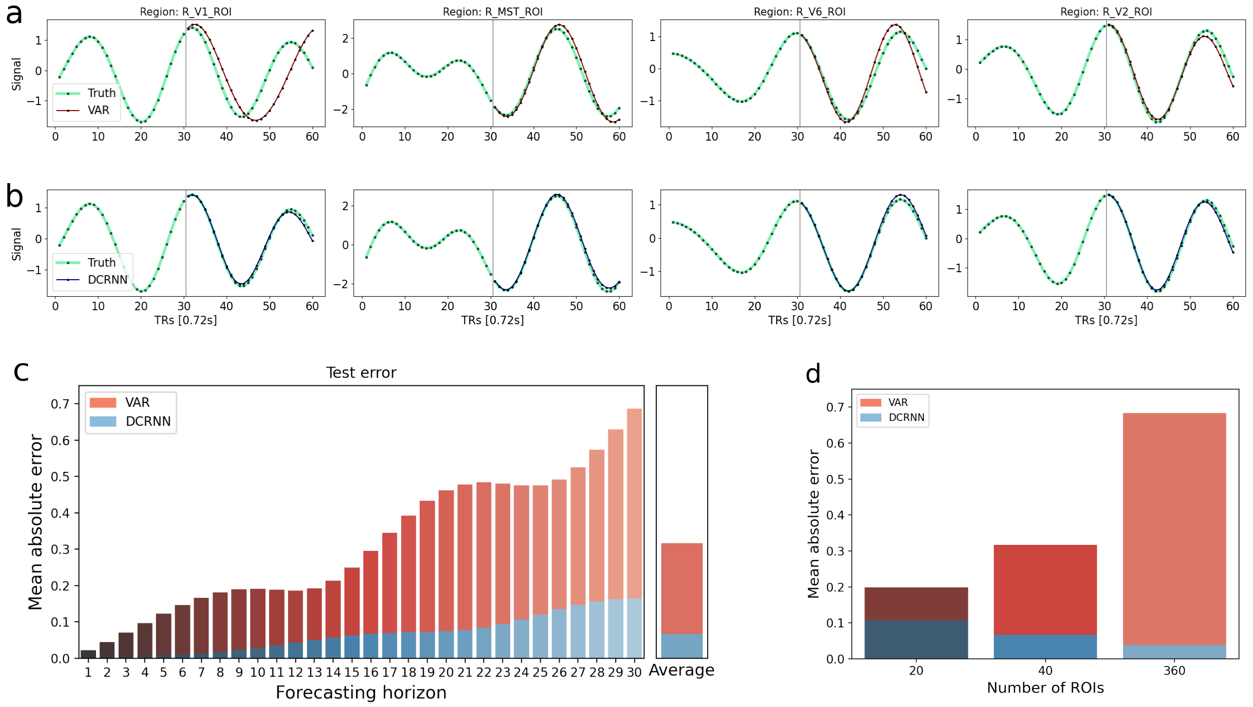

Figure S5: The figure illustrates the prediction accuracy on the UR dataset of a VAR model (a) in comparison to the DCRNN (b) using a network size of $N = 40$ ROIs. This illustrative example was chosen to be representative for the whole test set. The prediction error of the VAR model on this sample is with 0.305 slightly below average, while the error of the DCRNN is with 0.069 higher than its average. Below the average MAE over all samples in the test set is illustrated, in dependence of the forecasting horizon (c). In addition the overall test MAE in dependence of the brain network size is shown in (d), by including different number of ROIs in the network. Note that with increasing number of ROIs the MAE of the DCRNN decreases, whereas it increases for the VAR model.

In addition, in figure S5 (d) the overall test MAE of the VAR and DCRNN model in dependence of different network sizes N is depicted. We always selected the VAR model with order P which achieved the highest accuracy for the corresponding networksize N (with $P = 29$ for $N = 20$, $P = 30$ for $N = 40$ and $P = 20$ for $N = 360$). Figure S5 (d) illustrates that when more ROIs are included in the analysis, the accuracy of the VAR model degrades on this relatively small dataset, while in contrast the DCRNN model profits from learning the functional dynamics in more different regions. This points towards the advantage of learning localized filter on the structural network, as the number of parameters in the DCRNN are therewith independent of the network size N, and the predictions remain stable for large networks, even when only sparse imaging data is available.

Supplement IV

Here we reproduce the evaluations as described in subsection 'Model performance' employing a more liberal filtering of the fMRI timecourses. The preprocessing was carried out as described in subsection 'HCP data' but using a bandpass filter with cutoff frequencies $0.02 - 0.09\ Hz$ this time. The training of the two models was performed on 4 rs-fMRI sessions from 40 different subjects. To test the significance in the performance difference across subjects, the overall MAE was computed for every individual subject, as an average across sessions, brain regions and test samples. A paired t-test was applied and the difference in forecasting accuracy between the models was significant with $p \leq 0.0001$.

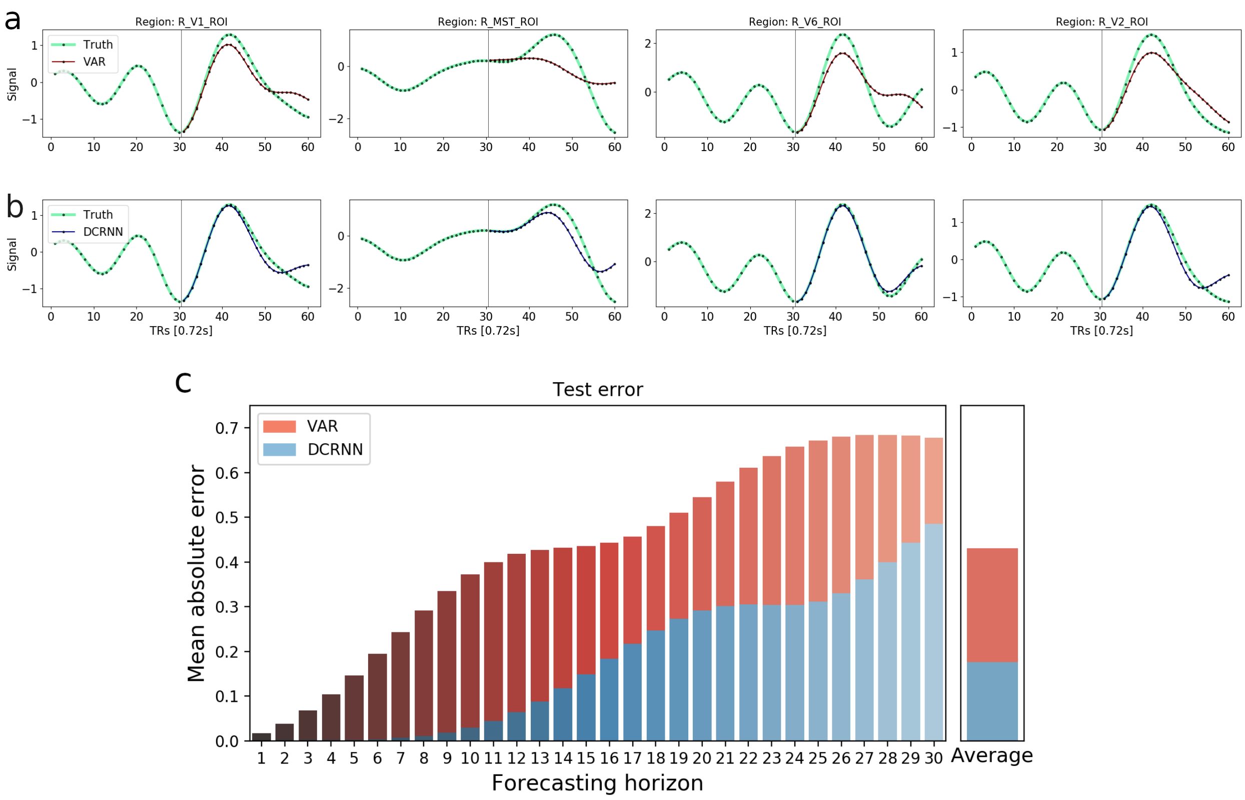

Figure S6: The figure illustrates the prediction accuracy of a VAR model (a) in comparison to the DCRNN (b) in the $0.02 - 0.09Hz$ frequency range. This illustrative example was chosen to be representative for the whole test set. The prediction error of the VAR model on this sample is with 0.428 slightly below average, while the error of the DCRNN is with 0.178 higher than its average. Below the average MAE over all samples in the test set is illustrated, in dependence of the forecasting horizon (c). On the right side in (c) the average of all horizons is shown.

Supplement V

In this section we replicate the evaluation from subsection 'Model performance', using a volumetric parcellation and applying an alternative method for probabilistic tractography of white matter tracks. Volumetric resting-state fMRI images provided by the HCP were subdivided in 90 cortical regions based on the automated anatomical labeling atlas (AAL) [10]. Timecourses within each region were averaged, and like in subsection 'Model performance', the $0.04 - 0.07Hz$ frequency band was selected for the analysis [4]. For the reconstruction of anatomical connectivity strengths, the multi-shell ball and stick model [1, 6] as implemented in FSL was employed [7]. Each region of the AAL atlas was defined as seed region, and probabilistic tractography (ProbtrackX) was run based on diffusion parameter estimation with BedpostX [1]. For each voxel 5000 samples were generated, and SC was quantified by counting how many streamlines starting in one region reached any other region of the AAL atlas. Those counts were normalized by dividing by the largest value, and an average SC matrix was computed across 10 different subjects. To test the significance in the performance difference across subjects, the overall MAE was computed for every subject again. Next a paired t-test was used and the difference in forecasting accuracy between the VAR and DCRNN model showed to be significant with $p \leq 0.0001$.

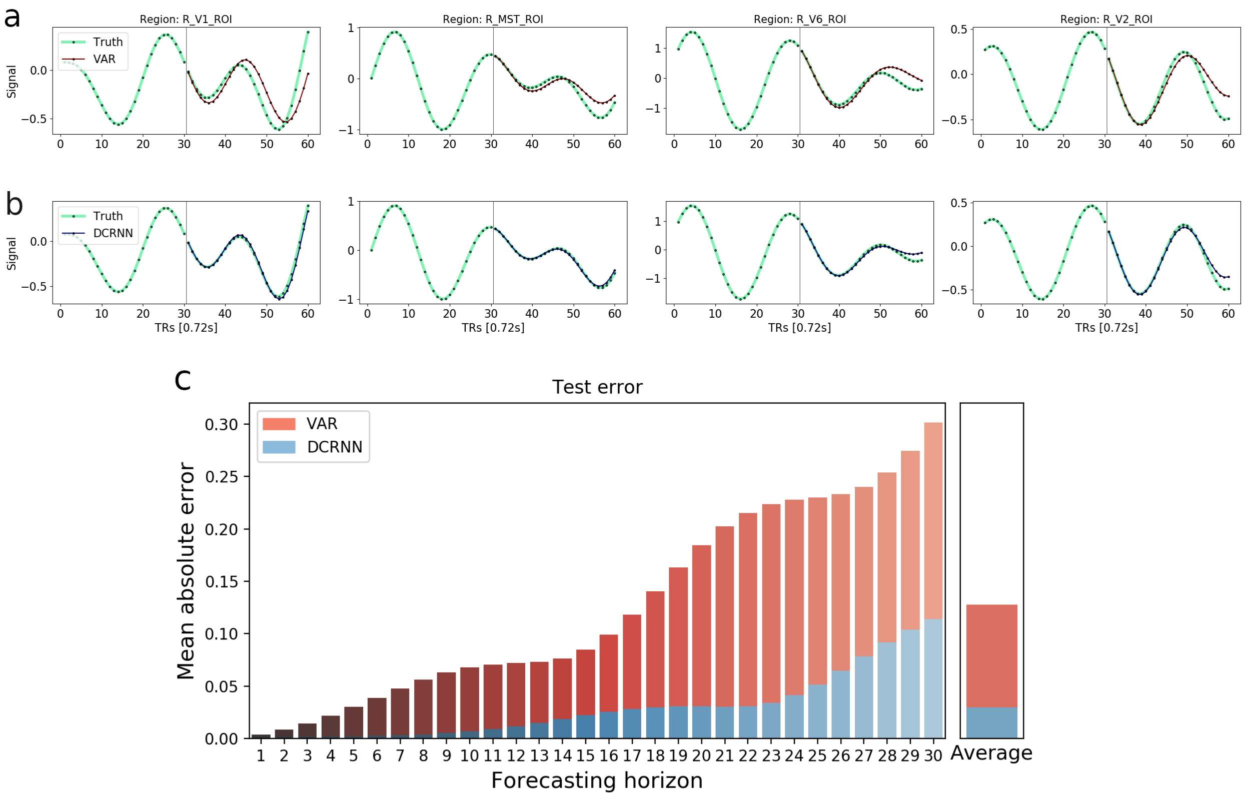

Figure S7: The figure illustrates the prediction accuracy of a VAR model (a) in comparison to the DCRNN (b). This illustrative example was chosen to be reasonable representative for the whole test set, the prediction error of the VAR model on this sample is with 0.119 slightly below average, while the error of the DCRNN is with 0.033 higher than its average. Below the average MAE over all samples in the test set is illustrated, in dependence of the forecasting horizon (c). On the right side in (c) the average of all horizons is shown.

Supplement VI

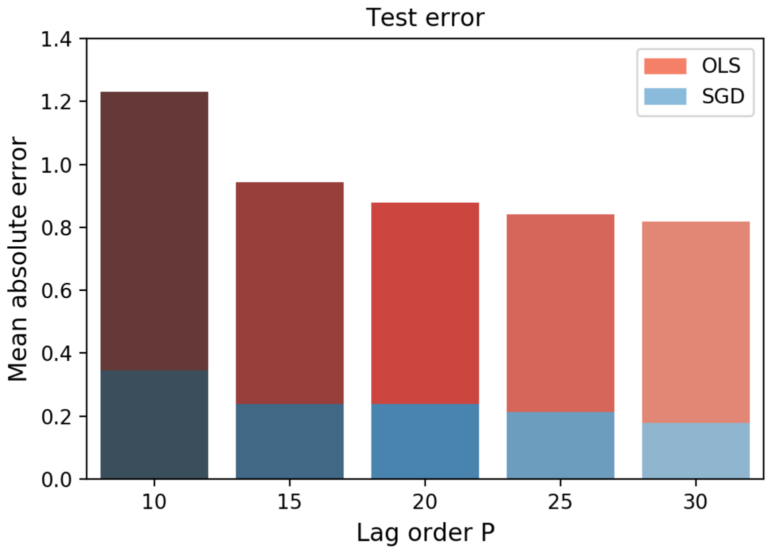

Figure S8: This figure shows the test MAE for two different optimization strategies to find the VAR coefficients, in dependence of lag orders P. The first one is performed with an ordinary least squares (OLS) fit on individual subject sessions and the average test error is depicted in red in this figure. The second one, in analogy to the training of the DCRNN, is based on gradient descent optimization, aggregating input-output pairs of samples across sessions like described in subsection 'Data description'. The test MAE of the stochastic gradient descent (SGD) approach is illustrated in blue.

References

[1] T. Behrens, H. Berg, S. Jbabdi, M. Rushworth, and M. Woolrich. Probabilistic diffusion tractography with multiple fibre orientations: What can we gain? *NeuroImage*, 34:144–55, 2007.

[2] J. Chung, C. Gulcehre, K. Cho, and Y. Bengio. Empirical evaluation of gated recurrent neural networks on sequence modeling, 2014.

[3] M. Glasser, T. Coalson, E. Robinson, C. Hacker, J. Harwell, E. Yacoub, K. Ugurbil, J. Andersson, C. Beckmann, M. Jenkinson, S. Smith, and D. Van Essen. A multi-modal parcellation of human cerebral cortex. *Nature*, 536, 2016.

[4] E. Glerean, J. Salmi, J. Lahnakoski, I. Jääskeläinen, and M. Sams. Functional magnetic resonance imaging phase synchronization as a measure of dynamic functional connectivity. *Brain connectivity*, 2:91–101, 2012.

[5] S. Hochreiter and J. Schmidhuber. Long Short Term Memory. *Neural Computation*, 9(8):1735–1780, 1997.

[6] S. Jbabdi, S. Sotiropoulos, A. Savio, M. Graña, and T. Behrens. Model-based analysis of multishell diffusion MR data for tractography: How to get over fitting problems. *Magnetic resonance in medicine : official journal of the Society of Magnetic Resonance in Medicine / Society of Magnetic Resonance in Medicine*, 68, 2012.

[7] M. Jenkinson, C. F. Beckmann, T. E. Behrens, M. W. Woolrich, and S. M. Smith. FSL. *NeuroImage*, 62(2):782 – 790, 2012.

[8] A. Puce, M. Latinus, A. Rossi, E. Dasilva, F. J. Parada, S. Love, A. Ashourvan, and S. Jayaraman. *Neural Bases for Social Attention in Healthy Humans*, pages 93–127. 2015.

[9] I. Sutskever, O. Vinyals, and Q. V. Le. Sequence to sequence learning with neural networks. *CoRR*, abs/1409.3215, 2014.

[10] N. Tzourio-Mazoyer, B. Landeau, P. DF, F. Crivello, O. Etard, N. Delcroix, B. Mazoyer, and J. Marc. Automated anatomical labeling of activations in spm using a macroscopic anatomical parcellation of the MNI MRI single-subject brain. *NeuroImage*, 15:273–89, 2002.
